# Soluble CD147 regulates endostatin via its effects on the activities of MMP-9 and secreted proteasome 20S

**DOI:** 10.3389/fimmu.2024.1319939

**Published:** 2024-01-22

**Authors:** Maya M. Rahat, Hala Sabtan, Elina Simanovich, Amir Haddad, Tal Gazitt, Joy Feld, Gleb Slobodin, Adi Kibari, Muna Elias, Devy Zisman, Michal A. Rahat

**Affiliations:** ^1^ Immunotherapy Laboratory, Carmel Medical Center, Haifa, Israel; ^2^ Department of Rheumatology, Carmel Medical Center, Haifa, Israel; ^3^ The Rappaport Faculty of Medicine, Technion-Israel Institute of Technology, Haifa, Israel; ^4^ Department of Rheumatology, Bnai Zion Medical Center, Haifa, Israel

**Keywords:** endostatin, CD147/EMMPRIN, MMP-9, secreted proteasome 20S, fibroblasts-monocytes interactions, angiogenesis

## Abstract

During progression of rheumatoid arthritis (RA), angiogenesis provides oxygen and nutrients for the cells’ increased metabolic demands and number. To turn on angiogenesis, pro-angiogenic factors must outweigh anti-angiogenic factors. We have previously shown that CD147/extracellular matrix metalloproteinase inducer (EMMPRIN) can induce the expression of the pro-angiogenic factors vascular endothelial growth factor (VEGF) and matrix metallopeptidase 9 (MMP-9) in a co-culture of the human HT1080 fibrosarcoma and U937 monocytic-like cell lines. However, whether CD147 influences anti-angiogenic factors was not known. We now show that relative to single cultures, the co-culture of these cells not only enhanced pro-angiogenic factors but also decreased the anti-angiogenic factors endostatin and thrombospondin-1 (Tsp-1), generally increasing the angiogenic potential as measured by a wound assay. Using anti-CD147 antibody, CD147 small interfering RNA (siRNA), and recombinant CD147, we demonstrate that CD147 hormetically regulates the generation of endostatin but has no effect on Tsp-1. Since endostatin is cleaved from collagen XVIII (Col18A), we applied different protease inhibitors and established that MMP-9 and proteasome 20S, but not cathepsins, are responsible for endostatin generation. MMP-9 and proteasome 20S collaborate to synergistically enhance endostatin generation, and in a non-cellular system, CD147 enhanced MMP-9 activity and hormetically regulated proteasome 20S activity. Serum samples obtained from RA patients and healthy controls mostly corroborated these findings, indicating clinical relevance. Cumulatively, these findings suggest that secreted CD147 mediates a possibly allosteric effect on MMP-9 and proteasome 20S activities and can serve as a switch that turns angiogenesis on or off, depending on its ambient concentrations in the microenvironment.

## 1 Introduction

Rheumatoid arthritis (RA) is characterized by synovial proliferation, leukocyte infiltration, inflammation, and progressive tissue damage. The increased rate of synovial hyper-proliferation and the increased metabolic needs of both the synovia and the infiltrating leukocytes result in local hypoxia, which is a potent driver of angiogenesis (1). Angiogenesis, the sprouting of new blood vessels from existing ones, is a critical process that provides adequate supply of oxygen and nutrients to joints and is associated with the growth and invasion of the pannus (2). Many pro-inflammatory mediators that are also pro-angiogenic are secreted by the infiltrating leukocytes and synovial fibroblasts that are found in the synovium, including cytokines [e.g., tumor necrosis factor α (TNFα), interleukin 6 (IL-6)], proteases [especially matrix metalloproteinases (MMPs)], and growth factors [e.g., vascular endothelial growth factor (VEGF), basic fibroblast growth factor (bFGF), transforming growth factor β (TGFβ)] (3).

Angiogenesis is regulated by a precise balance between pro-angiogenic factors (e.g., MMP-9, VEGF) and anti-angiogenic factors [e.g., thrombospondin-1 (Tsp-1), endostatin]. MMPs and other proteases can break down the basement membranes and extracellular matrix (ECM) in the perivascular space to allow proliferation and movement of endothelial cells (4), while VEGF and other growth factors activate endothelial cells and attract leukocytes to the site (5). Tsp-1, endostatin, and other anti-angiogenic factors inhibit the proliferation and migration of endothelial cells (6–8). The tissue inhibitors of metalloproteinases (TIMPs), particularly TIMP-1, which is more specific to MMP-9, are implicated mostly as anti-angiogenic factors (9, 10). When the concentrations of pro-angiogenic factors exceed those of anti-angiogenic factors, the balance is disrupted and the “angiogenic switch” is turned on, resulting in the activation of the endothelial cells and the onset of angiogenesis (2).

Extracellular matrix metalloproteinase inducer (EMMPRIN/CD147) is a multifunctional transmembranal protein that mediates interactions between epithelial cells, fibroblasts, and immune cells (11). Although it has been studied mostly in tumor cells, it is now recognized as pro-inflammatory and a pro-angiogenic factor in autoimmune diseases such as RA (12–15). Mechanistically, CD147 enhances angiogenesis through homophilic binding with another CD147 molecule that induces the production of VEGF and MMPs, including MMP-9 (11, 16, 17), as well as by directly regulating endothelial cells by enhancing their expression of VEGF receptor II (VEGFRII) (18) and acting as its co-receptor (19). Secreted forms of CD147 have also been shown to be functional and can induce the secretion of both MMP-9 and VEGF in a co-culture system of human fibroblasts and monocytic-like cell lines (20, 21).

MMPs and other proteases can cleave different ECM proteins to generate anti-angiogenic factors. For example, MMP-9 cleaves collagen IV to generate tumstatin; MMP-2, MMP-7, and MMP-9 can cleave plasmin to generate angiostatin; and MMP-7 and MMP-9 can cleave collagen XVIIIA (Col18A) to generate endostatin (22–25). Endostatin is a 22-kDa cleavage product of Col18A, which is a heparan sulfate proteoglycan found in various epithelial and vascular basement membranes (BMs) (26). Col18A, a member of the multiplexin family, has a structure of non-triple-helical regions or non-collagenous (NC) regions interrupting several triple-helical domains that allow flexibility of the protein (27). Col18A contains 10 triple helical collagenous domains (Col1–10) that are flanked by 11 non-collagenous regions (NC1–11). The NC1 domain contains a short region that mediates the homotrimerization of the protein, a hinge domain that is sensitive to different proteases, and a C-terminus domain that upon cleavage releases endostatin (25, 27). Many proteases can cleave the 38-kDa NC1 domain to form endostatin or endostatin-containing fragments with different molecular weights. These include cathepsin L and cathepsin S, elastase, and MMP-7 as well as MMP-9, MMP-14, and MMP-2 (25, 28, 29). Recently, the secreted form of proteasome 20S was suggested to be involved in generating endostatin (30). The generated endostatin is considered a potent anti-angiogenic factor, as it inhibits endothelial cell proliferation and migration by binding to the three VEGF receptors on endothelial cells and inhibiting VEGF signaling (6).

Because the pro-angiogenic CD147 can induce VEGF and MMPs and different MMPs can cleave Col18A to generate endostatin, we hypothesized that CD147 has a role in the regulation of anti-angiogenic factors such as endostatin. We show that this is indeed the case, and CD147 indirectly regulates endostatin by oppositely regulating the activities of MMP-9 and secreted proteasome 20S.

## 2 Materials and methods

### 2.1 Cultured cells

The human fibrosarcoma cell line HT1080 (ATCC CCL-12012) was cultured in Dulbecco’s modified Eagle’s medium (DMEM; Biological Industries, Beit He’emek, IL), with 10% fetal calf serum (FCS), L-glutamine (2 mM), amphotericin B (2.5 μg/ml), non-essential amino acids (NEAAs; 0.1 mM each of alanine, asparagine, aspartic acid, glutamic acid, glycine, proline, and serine), and 1% antibiotics (penicillin 0.016 mM, streptomycin 0.15 mM, neomycin 0.11 mM). To support their growth, the medium was supplemented with 25% conditioned medium obtained from the human promyelocytic leukemia cells HL60 (ATCC CRL-240) that contains secreted FGF-2. The U937 monocyte-like cells (ATCC CRL-1593) were cultured in RPMI-1640 medium with 10% FCS, L-glutamine (2 mM), 1% amphotericin B, and the same antibiotics. The human endothelial cell line EaHy926 (ATCC CRL-2922) was cultured in DMEM with 10% FCS, glutamine (2 mM), 2% HAT (hypoxanthine 0.1 mM, aminopterin 0.4 mm, thymidine 16 mm), amphotericin B (2.5 μg/ml), and 1% same antibiotics. All cell lines were split twice a week at a ratio of 1:3 or 1:4. All cell lines were regularly monitored for morphological changes and tested for the presence of mycoplasma.

HT1080 cells were incubated overnight in 24- or 96-well plates (3 × 10^5^ cells/800 μl or 3 × 10^4^ cells/100 μl, respectively), and after cells adhered to the plate, the medium was replaced with starvation medium [HT1080 medium without FCS, supplemented with 0.1% bovine serum albumin (BSA)], to avoid possible masking of signals initiated by exogenous stimuli. For the co-culture experiments, inserts (0.4-μm pore size) were used to prevent cell–cell contact and allow RNA and protein extraction from each cell line. HT1080 cells were seeded in the lower chamber and allowed to adhere, and the U937 cells were plated in the upper chamber at a ratio of 1:1. The strong MMP-9 inducer TNFα (1 ng/ml) was added. Some experiments included the addition of the rabbit anti-human CD147 antibody (h161-pAb, 2 ng/ml, produced in our lab), the human recombinant CD147 protein (R&D Systems, Minneapolis, MN, USA), or the human recombinant TIMP-1 (R&D Systems). In other experiments, the human recombinant MMP-9 (R&D Systems) and the human erythrocyte-derived 20S proteasome protein (R&D Systems) were added to the culture. Other experiments included the addition of different protease inhibitors (**Supplementary Table S1**). After 48 h of incubation, supernatants were collected and frozen immediately at −80°C for further analysis of the concentrations of different factors.

### 2.2 Sandwich enzyme-linked immunosorbent assay (ELISA)

To determine the concentrations of the pro- and anti-angiogenic factors, human DuoSet ELISA kits for CD147, MMP-9, MMP-2, VEGF, endostatin, thrombospondin-1, TIMP-1, and TIMP-2 were used according to the manufacturer’s instructions (R&D Systems). Serum samples or conditioned media were diluted in dilution buffer [1% BSA in phosphate-buffered saline (PBS)] to 1:100 for CD147, MMP-9, VEGF, TIMP-1, TIMP-2, and MMP-2; 1:20 for endostatin; and 1:1,000 for Tsp-1, according to previous calibration experiments.

### 2.3 Knocking down CD147 expression

Two CD147-designed small interfering RNA (siRNA) molecules (10 nM each) or an siRNA negative control (NC; 10 nM) was mixed with Lipofectamine RNAiMAX (Thermo Fisher Scientific, Waltham, MA) that was diluted 1:25 in Opti-MEM medium and spread on each well. The HT1080 cells (10^5^ cells) were added and incubated for 24 h in full medium without antibiotics. Then, the medium was replaced with serum-starvation medium with 0.1% BSA and TNFα (1 ng/ml), and cells were co-cultured for 48 h with U937 cells at a ratio of 1:1 using inserts with a 0.4-μm pore size, to prevent cell–cell contact.

### 2.4 Wound assay

The human endothelial cell line EaHy926 (2 × 10^4^ cells) was seeded in 96-well plates and incubated overnight to confluency. Then, cells were scratched with the edge of a 200-μl tip, the detached cells were washed away with 1× PBS, and the line of injury was marked. The remaining endothelial cells were then incubated with 100 μl of supernatants (diluted 1:2 with EaHy926 full medium) derived either from co-cultured or HT1080 cell line alone and with or without h161-pAb (2 ng/ml). Alternatively, the endothelial cells were incubated with serum samples from healthy volunteers and RA patients (diluted 1:2 with the EaHy926 full medium), with and without the addition of anti-CD147 (2 ng/ml). To show the natural rate of wound healing and closure of the gap by the endothelial cells themselves, EaHy926 cells were incubated without any additions in full medium. Images of the field of injury were acquired immediately after the wound was applied (0 h) and at the end of the experiment (20 h) at magnification of ×4. The average distance that the endothelial cells migrated to in order to close the gap was determined by subtracting the distance between the two sides of the scratch at 20 h from the distance at 0 h, using the Image-Pro Plus 4.5 software (Media Cybernetics, Inc., Rockville, MD).

### 2.5 Quantitative real-time PCR (qPCR)

Total RNA was extracted from cells using a total RNA purification kit (Norgen Biotek, Thorold, Canada), according to the manufacturer’s instructions. Total RNA (1,000 ng) was transcribed to complementary DNA (cDNA) using the High Capacity cDNA Reverse Transcription kit (Thermo Fisher Scientific/Applied Biosystems, Waltham, MA) at 37°C for 1 h. Expression of *collagen XVIIIA1*, and the reference gene *PBGD* was quantified by real-time PCR using the EvaGreen assay kit (Solis BioDyne, Tartu, Estonia). The list of the primers can be found in **Supplementary Table S2**. The reactions used 80 ng of transcribed cDNA and were carried out for 40 cycles, each of 15 s at 95°C and 60 s at 60°C, using the StepOne real-time PCR system (Thermo Fisher Scientific/Applied Biosystems). The comparative method (2^−ΔΔCT^) was used for relative quantification, where untreated cells were used as calibrators.

### 2.6 Western blot analysis

Equal amounts of ECM proteins (15 μg/lane) were extracted from the wells using radioimmunoprecipitation assay (RIPA) buffer and were loaded onto a 10% sodium dodecyl sulfate–polyacrylamide gel electrophoresis (SDS-PAGE) gel. After electrophoretic separation, proteins were transferred onto pre-cut nitrocellulose membranes (Advansta, San Jose, CA, USA), which were then blocked with Block-Chemi blocking solution (Advansta) overnight at 4°C. The membranes were incubated for 1 h at room temperature with the primary anti-endostatin antibody (R&D Systems) diluted 1:3,000 in blocking solution, and after three washes with the wash buffer [1× Tris-buffered saline (TBS) with 0.05% Tween 20], they were incubated with a horseradish peroxidase (HRP)–conjugated secondary antibody [donkey anti-goat immunoglobulin G (IgG), Jackson ImmunoResearch Labs, West Grove, PA, USA] diluted 1:5,000 in blocking buffer. After three additional washes, membranes were incubated with the WesternBright ECL HRP substrate (Advansta), and protein bands were visualized using the Omega Lum G imaging system.

### 2.7 MMP-9 activity assay

As the MMP-9 substrate can also be cleaved by MMP-2, 96-well ELISA plates were first coated with a capture antibody specific for MMP-9 (2 μg/ml, R&D Systems), and after overnight incubation, it was washed with 1× PBS. Supernatants (15 μl) derived from the experimental samples were incubated in the plate for 1 h, and then the plate was washed three times with 1× PBS. The captured MMP-9 was then incubated with 50 μM of the MMP-9 fluorescent substrate DNP-PLGMWSR (trifluoroacetate salt, Cayman, Ann Arbor, MI, USA) and the assay buffer (50 mM tricine, 0.2 M NaCl, 10 mM CaCl_2_, and 0.05% Brij-35) for 5 min. The supernatant was then transferred to a black 96-well plate, and the fluorescence emitted by the cleaved peptide was read at 490-nm excitation and 520-nm emission. As positive control, we used recombinant MMP-9 that was activated overnight with aminophenylmercuric acetate (APMA; 1 mM, Merck/Sigma-Aldrich, Darmstadt, Germany).

### 2.8 Proteasome 20S activity assay

Supernatants (15 μl) derived from HT1080, U937, or their co-culture were diluted 1:8 with the proteasome 20S assay buffer [3.8 mM ethylenediaminetetraacetic acid (EDTA), 25 mM 4-(2-hydroxyethyl)-1-piperazineethanesulfonic acid (HEPES) buffer, 9.5 mM dithiothreitol (DTT)] and incubated with 15 μM of the proteasome 20S fluorescent substrate SUC-LLVY-2R110 (AAT Bioquest, Sunnyvale, CA, USA) for 5 min in a black 96-well plate. The fluorescence emitted by the cleaved peptide was read at 490-nm excitation and 520-nm emission. As positive control, proteasome 20S preparation protein was activated for 1 h with 0.5% SDS.

### 2.9 Production of decellularized extracellular matrix (ECM)

HT1080 cells (3 × 10^4^ cells/100 μl) were seeded in a 96-well plate overnight in full medium. After an overnight incubation, the medium was replaced with HT1080 starvation medium containing TNFα (1 ng/ml), and the cells were incubated for an additional 72 h. At the end of the incubation, supernatants were removed, and cells were destroyed by incubating them with hypotonic double-distilled water (DDW) for 20 min. Then, the cell debris was washed away three times with 1× PBS, resulting in decellularized ECM that coated the plate and contained collagen XVIIIA among other ECM proteins. Supernatants derived from the experimental samples were then added to the ECM together with increasing concentrations of either MMP-9 inhibitor I, the proteasome 20S inhibitor AM-114, or recombinant MMP-9 (R&D Systems) that was activated with APMA (1 mM, for 24 h) or with a proteasome 20S preparation.

### 2.10 Study population

A total of 20 RA patients with active disease were recruited from the rheumatology clinics in Carmel Medical Center and in Bnai Zion Medical Center in Haifa, Israel. All patients met the 2010 American College of Rheumatology/European League Against Rheumatism (ACR/EULAR) RA criteria. Patients with diagnosis of another inflammatory arthritis or neoplastic disease were excluded. Parameters were collected at baseline and included demographic data, height, weight, calculated body mass index (BMI) (weight/height^2^), patient comorbidities, current medications, and physical examination data with a focus on swollen and tender joint counts. Laboratory variables included complete blood count and chemistry panel, lipid panel, C-reactive protein (CRP), and rheumatoid factor (RF), all analyzed by standard methods in a central laboratory. These data are summarized in **Table 1**. Serum samples were obtained for further analysis of the pro- and anti-angiogenic factors.

**Table 1:** Demographic and clinical characteristics, underlying diseases, and treatment of the study and control groups.

	RA group	Control group	P-value
Number of participants	20	25	–
Sex: female (%)	17 (85%)	19 (95%)	ns
Age (years) ± SD	65.1 ± 6.05	51.44 ± 2.10	0.0007
BMI ± SD	27.5 ± 1.09	26.56 ± 1.19	ns
Disease duration (years) ± SD	9.9 ± 1.9	–	–
Tobacco use (%)	4 (20%)	3 (15%)	ns
Alcohol use (%)	0 (0%)	7 (35%)	0.0123
RF (%)	17 (40%)	0 (0%)	0.0017
CRP ± SD	1.534 ± 0.33	–	–
Comorbidities			
Hypertension (%)	10 (50%)	–	–
Hyperlipidemia (%)	5 (25%)	–	–
Diabetes mellitus (%)	4 (20%)	–	–
**Medication (at baseline)**		–	–
Methotrexate (MTX)	20 (100%)	–	–
Leflunomide (LEF)	1 (5%)	–	–
Sulfasalazine (SSZ)	1 (5%)	–	–
Hydroxychloroquine (Plaquenil-PLQ)	3 (15%)	–	–
Corticosteroid (%)	3 (15%)	–	–

ns, not significant.

The control group of 25 volunteers without inflammatory diseases, renal failure, cardiovascular disease (CVD), or known malignancies was age- and gender-matched with the RA group. Blood samples from this group were drawn at recruitment and tested for the pro- and anti-angiogenic factors.

The study protocol was approved by the local institutional review boards of the participating medical centers, and informed consent was obtained from all participants at recruitment (approval number at Carmel Medical Center CMC-0130-17; approval number at Bnai Zion Medical Center 0142-17-BNZ).

### 2.11 Statistical analysis

All values are presented as means ± SEM (standard error of the mean), and the number of biological repetitions is indicated in the legend of each figure (n). Two groups were compared using the unpaired two-tailed Student’s *t*-test. Multiple groups were compared using one-way analysis of variance (ANOVA) followed by Bonferroni’s *post-hoc* test, and two parameters in multiple groups were compared using two-way ANOVA and Bonferroni’s *post-hoc* test. The patient’s data were analyzed using a two-tailed Mann–Whitney U-test when two groups were compared. P-values exceeding 0.05 were not considered significant.

## 3 Results

### 3.1 The co-culture promotes angiogenesis

To explore the hypothesis that CD147 can regulate anti-angiogenic factors as well as pro-angiogenic factors, we first established a co-culture model by incubating the human fibrosarcoma cell line HT1080 with the U937 monocytic-like cell line, using inserts to prevent cell-to-cell contact. As controls, each cell line was incubated alone. Since MMPs, in particular MMP-9, are important pro-angiogenic factors, we added to all experimental groups the strong MMP inducer, TNFα, in a minimal concentration (1 ng/ml) that strongly induced MMP-9 but did not cause cell death, as previously described (20). This co-culture system allowed us to simulate the interactions between the two relevant cell types in the synovial space, fibroblasts and monocytes, and retain the ability to extract RNA and protein from each cell line separately.

**Figure 1:**
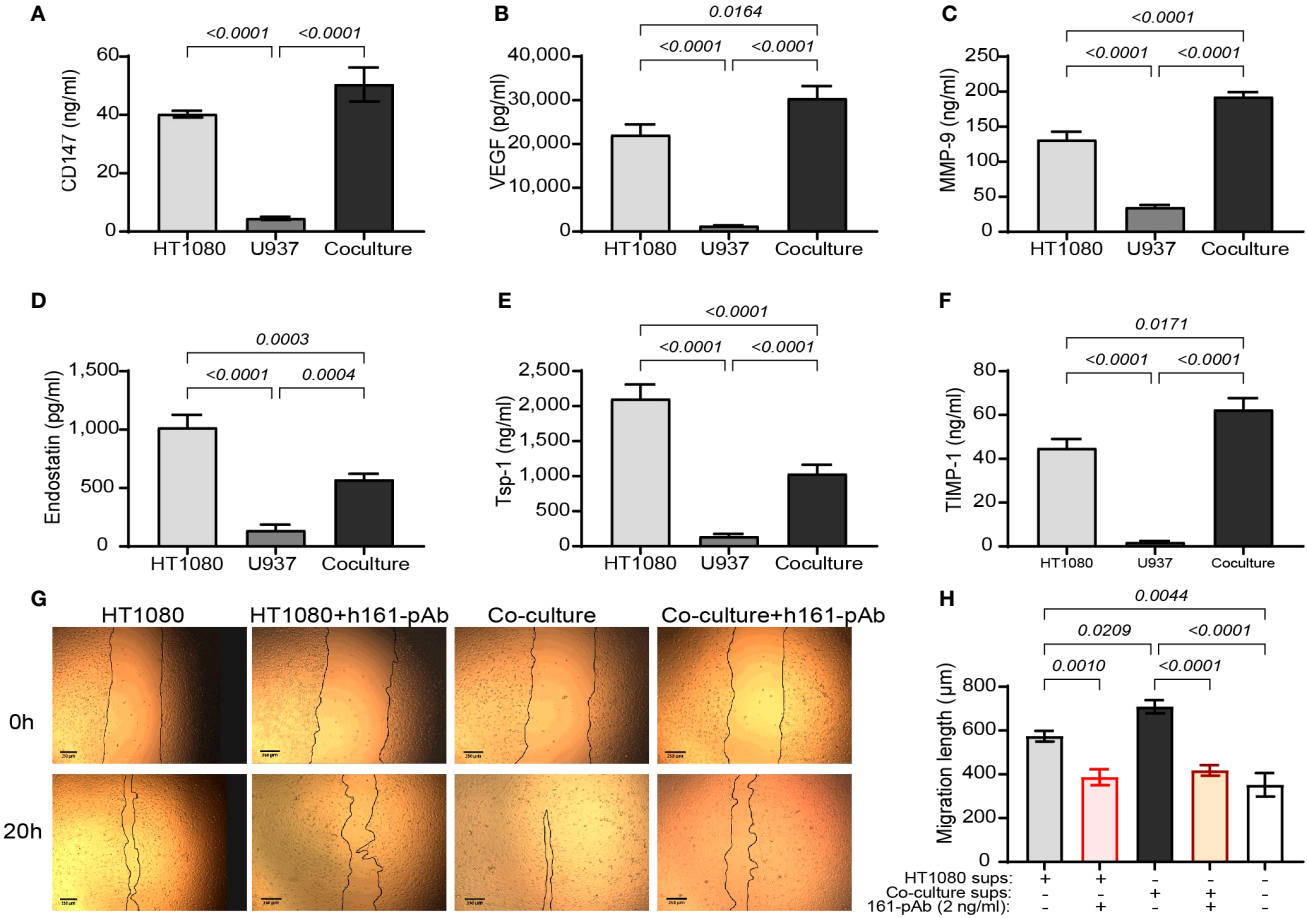
The angiogenic potential of the co-culture. The human HT1080 fibroblast cell line (3 × 10^5^ cells) or the human U937 monocytic-like cell line (3 × 10^5^ cells) were plated in 24-well plates alone or in co-culture (U937 cells were plated in inserts with 0.4-μm pores size) and incubated for 48 h in the presence of TNFα (1 ng/ml). Then, supernatants were collected, and the concentrations of **(A)** CD147, **(B)** VEGF, **(C)** MMP-9, **(D)** endostatin, **(E)** Tsp-1, and **(F)** TIMP-1 were assessed by ELISA (n = 8). Data are presented as means ± SE and were analyzed using one-way ANOVA followed by Bonferroni’s *post-hoc* test. **(G, H)** EaHy926 cells (2 × 10^4^ cells) were grown to confluency, and then a scratch was applied using the edge of a tip. Detached cells were washed away, and supernatants derived from the experimental groups were diluted 1:2 with serum-starvation medium and applied onto the endothelial layer, with or without the presence of the rabbit anti-human CD147 antibody (h161-pAb, 2 ng/ml). Images were taken at the beginning of the experiment (0 h) and after 20 h. **(G)** Representative images and **(H)** the measured distance between the two sides of the scratch at 20 h subtracted from the distance at 0 h (n = 8–9 per group) are shown. Data are presented as means ± SE and were analyzed using one-way ANOVA followed by Bonferroni’s *post-hoc* test.

To examine the overall angiogenic potential of the co-culture compared to that of each cell line alone, we first determined the concentrations of both pro- and anti-angiogenic factors in the supernatants. Relative to each of the single cultures, the concentrations of VEGF, MMP-9, and TIMP-1 in the co-culture were increased (by 1.5-, 1.4-, and 1.3-fold, respectively, **Figures 1B, C, F**). In contrast, a reverse effect was evident for endostatin and Tsp-1, and their concentrations were decreased in the co-culture relative to those in the HT1080 single-cell culture (both by 55%, p < 0.05, **Figures 1D, E**). Surprisingly, CD147 concentrations in the co-culture were not significantly changed relative to those in the HT1080 single culture (**Figure 1A**).

To further show that the balance between pro- and anti-angiogenic factors in the co-cultures turns on the angiogenic switch, the angiogenic potential of the supernatants on the migration and proliferation of the human endothelial cell line EaHy926 cells was evaluated by the wound assay. The supernatants from the co-culture enhanced the rate of migration and the closure of the gap made by the scratch, relative to those of the single culture of HT1080 cells. Not surprisingly, the addition of the anti-CD147 antibody h161-pAb slowed this down (**Figures 1G, H**), providing the first evidence that CD147 regulates the angiogenic potential of the cells. We therefore concluded that the co-culture enhanced the angiogenic potential of the cells and that the co-culture can be used as a model system to investigate the mechanisms at play.

### 3.2 CD147 regulates the anti-angiogenic factor endostatin, but not Tsp-1

CD147 has been shown to enhance angiogenesis via its ability to induce pro-angiogenic factors such as VEGF and MMPs, but its effects on anti-angiogenic factors are unknown (11, 18, 31). To test whether CD147 is involved in the regulation of anti-angiogenic factors, we used our co-culture model in three different approaches: neutralizing CD147 with the anti-CD147 antibody, knocking down its expression in HT1080 fibroblasts, and adding exogenous recombinant CD147.

**Figure 2:**
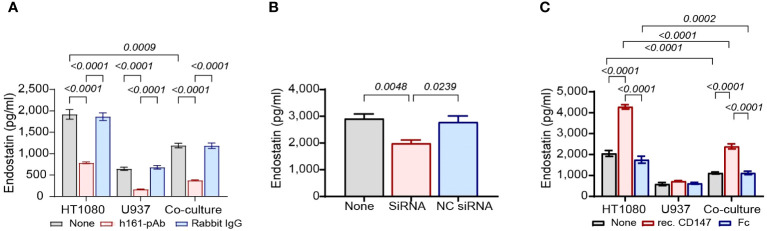
CD147 regulates the accumulation of endostatin. HT1080 cells (3 × 10^4^ cells) were incubated alone or in co-culture with U937 cells (3 × 10^4^ cells) for 48 h in the presence of TNFα (1 ng/ml). **(A)** The cells were incubated alone, with the anti-CD147 antibody (h161-pAb, 2 ng/ml) or with an irrelevant antibody (rabbit IgG, 2 ng/ml). After 48-h incubation, supernatants were collected, and the concentrations of endostatin (n = 6) were determined by ELISA. Data are presented as means ± SE and were analyzed using two-way ANOVA followed by Bonferroni’s *post-hoc* test. **(B)** The human HT1080 fibroblast cell line (10^5^ cells) was transfected with two CD147 siRNA molecules (10 nM each) and left for 24 h in full medium. Then, the medium was replaced with serum-starvation medium (antibiotics-free) with TNFα (1 ng/ml), and U937 cells (10^5^ cells) were added in inserts (0.4-μm pore size). After 48-h incubation in co-cultures, supernatants were collected, and the concentrations of endostatin (n = 8) were assessed using ELISA. Data are presented as means ± SE and were analyzed using one-way ANOVA followed by Bonferroni’s *post-hoc* test. **(C)** HT1080 cells (3 × 10^4^ cells), U937 cells (3 × 10^4^ cells), and their co-culture were incubated in serum-starvation medium with TNFα (1 ng/ml), in the presence of the human recombinant CD147 (300 ng/ml) or the Fc fragment (300 ng/ml) for 48 h. After incubation, supernatants were collected, and the concentrations of endostatin (n = 6) were assessed by ELISA. Data are presented as means ± SE and were analyzed using two-way ANOVA followed by Bonferroni’s *post-hoc* test.

First, we neutralized CD147 by adding the rabbit anti-human CD147 antibody (human polyclonal 161-Ab, 2 ng/ml) and measured its effects on the levels of both pro-angiogenic (MMP-9, VEGF, and TIMP-1) and anti-angiogenic (endostatin and Tsp-1) factors in supernatants. As controls, we used the non-specific rabbit IgG. We show that the co-culture inhibited the accumulation of endostatin by 38% [1,918 ± 113 pg/ml and 1,190 ± 54 pg/ml in HT1080 cells (gray leftmost bar) and co-culture (gray rightmost bar), respectively] and that the addition of h161-pAb inhibited the accumulation of endostatin by 59% relative to that in the untreated HT1080 cells (1,918 ± 113 pg/ml vs. 787 ± 21 pg/ml) (**Figure 2A**). We also show that h161-pAb inhibited the pro-angiogenic factors MMP-9 and VEGF compared to the untreated single culture, as was expected and previously shown by us (**Supplementary Figures S1A, B**) (20). On the other hand, h161-pAb increased the accumulation of TIMP-1 (**Supplementary Figure S1D**) and did not affect the accumulation of Tsp-1 (**Supplementary Figure S1C**).

Next, we knocked down CD147 expression in HT1080 using siRNA specifically designed for CD147, or NC-siRNA as negative control, and then co-cultured these cells with U937 monocytes. The siRNA successfully knocked down CD147 expression, as CD147 expression was reduced by 79.3% (44.8 ± 3.3 ng/ml in untreated HT1080 cells, gray bar, vs. 9.3 ± 1.6 ng/ml in CD147 siRNA–infected HT1080 cells, red bar, **Supplementary Figure S1E**). In the co-culture, the reduced CD147 expression caused a 32% reduction in the accumulation of endostatin (2,912 ± 173 pg/ml in untreated co-culture, gray bar, vs. 1,989 ± 121 pg/ml in CD147 siRNA–infected HT1080 in co-culture, red bar, **Figure 2B**) and significantly reduced the expression of the pro-angiogenic factors VEGF and MMP-9 relative to that in the untreated co-cultured cells (**Supplementary Figures S1F, G**). However, the levels of TIMP-1 were only modestly increased (**Supplementary Figure S1I**), whereas those of Tsp-1 remain unchanged (**Supplementary Figure S1H**).

**Figure 3:**
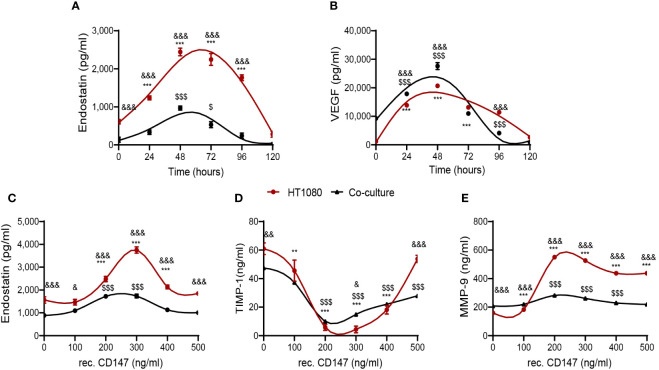
CD147 hormetically regulates endostatin. The HT1080 fibroblast cell line (3 × 10^5^ cells) was cultured alone or co-cultured with U937 cells (3 × 10^5^ cells) in serum-starvation medium in the presence of TNFα (1 ng/ml). **(A, B)** Supernatants were collected at different time points, and the concentrations of **(A)** endostatin (n = 6) and **(B)** VEGF (n = 6) were assessed by ELISA. Data are presented as means ± SE and were analyzed using two-way ANOVA followed by Bonferroni’s *post-hoc* test. **(C–E)** Increasing concentrations of recombinant CD147 were added, and the cultures were incubated for 48 h. Supernatants were collected, and concentrations of **(C)** endostatin, **(D)** TIMP-1 (n = 6), and **(E)** MMP-9 (n = 6) were assessed using ELISA. **, p < 0.01; ***, p < 0.001 relative to unstimulated HT1080 cells; $, p < 0.05, $$$, p < 0.001 relative to unstimulated co-culture; &, p < 0.05, &&, p < 0.01, &&&, p < 0.001 relative to HT1080 cells in each concentration. Data are presented as means ± SE and were analyzed using two-way ANOVA followed by Bonferroni’s *post-hoc* test.

Finally, we incubated the co-culture and each of the single cultures with exogenous human recombinant CD147 at a concentration of 300 ng/ml, which was found to be the most effective (**Figure 3C**). Since this protein is fused to the Fc fragment of IgG, we used the purified Fc fragment as negative control at the same concentration. Recombinant CD147 increased the anti-angiogenic factor endostatin by twofold in both single cultures and co-cultures relative to those in the untreated groups (4,279.5 ± 107 pg/ml vs. 2,053 ± 142 pg/ml in the single culture, 2,388 ± 121 pg/ml vs. 1,113 ± 51 pg/ml in the co-culture, **Figure 2C**). Recombinant CD147 also enhanced VEGF and MMP-9 (**Supplementary Figures S1J, K**), decreased the levels of TIMP-1 (**Supplementary Figure S1M**), and had no impact on the expression of Tsp-1 (**Supplementary Figure S1L**).

Thus, we conclude that CD147 is involved not only in the regulation of pro-angiogenic factors, through its ability to induce VEGF and MMP-9, but also in the regulation of the anti-angiogenic factor endostatin. In other words, CD147 is responsible for the balance between these pro- and anti-angiogenic factors and therefore may be a key factor in the activation of the angiogenic switch.

### 3.3 CD147 regulates endostatin in a hormetic manner

The finding that CD147 induces the pro-angiogenic factors VEGF and MMP-9, as well as the anti-angiogenic factor endostatin, raised the question whether CD147 could both promote angiogenesis and inhibit it simultaneously. One possibility is that the kinetics of the opposing factors are different, and another possibility is that different concentrations of CD147 have different effects on these factors.

First, we examined the kinetics of the accumulation of endostatin and VEGF (**Figures 3A, B**) and observed a similar pattern, where the peak of concentrations was reached after 48 h, both in single HT1080 cultures and in the co-cultures, ruling out the first possibility. Next, we examined the effects of the addition of different concentrations of recombinant CD147 on the accumulation of endostatin, TIMP-1, and MMP-9. Endostatin reached a peak at CD147 concentrations of 250–300 ng/ml and then declined, exhibiting a bell-shaped hormetic response (**Figure 3C**). Similarly, the known MMP-9 inhibitor TIMP-1 demonstrated a U-shaped graph, reaching a nadir at the same CD147 concentrations of 200–300 ng/ml (**Figure 3D**), representing a hormetic response with an opposite trend. In contrast, the effect of CD147 on MMP-9 accumulation was not hormetic, as concentrations above 300 ng/ml only moderately inhibited MMP-9 accumulation (**Figure 3E**).

### 3.4 Endostatin is reduced in the co-culture posttranscriptionally and posttranslationally

**Figure 4:**
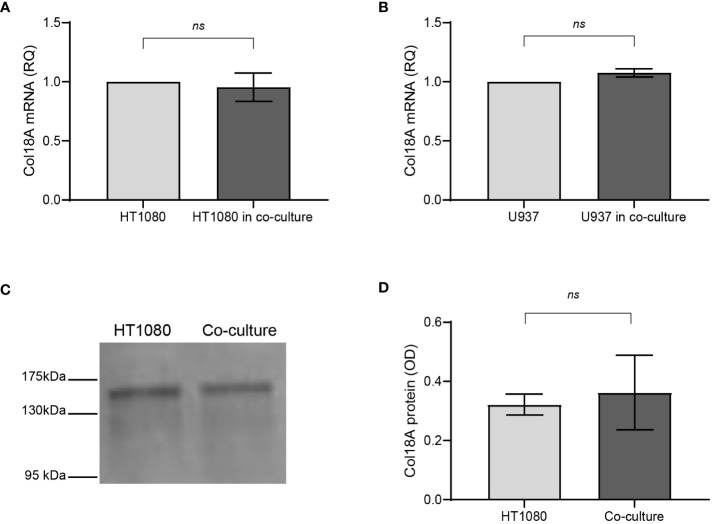
Endostatin is regulated in co-culture posttranslationally. HT1080 cells (3 × 10^6^ cells) were incubated alone or in co-culture with U937 cells (3 × 10^6^ cells) in serum-starvation medium for 48 h and in the presence of TNFα (1 ng/ml), separated by inserts (0.4-μm pore size) to allow separate extraction of RNA from each cell type. Supernatants were collected, and total RNA was extracted, transcribed to cDNA, and amplified by qPCR using primers specific for Col18A (n = 8). **(A)** Col18A mRNA in HT1080 cells in single cultures and co-cultures. **(B)** Col18A mRNA in U937 cells in single cultures and co-cultures (n = 8). Data are presented as means ± SE and analyzed using the two-tailed unpaired Student’s *t*-test. **(C)** A representative image of a Western blot of the ECM extracted from the plates, probed with an anti-endostatin antibody that also detects the precursor Col18A, and **(D)** its quantitation (n = 3). ns, not significant.

CD147 is a transmembranal protein that can transduce signals that activate mitogen-activated protein kinases (MAPKs) and phosphoinositide 3-kinase (PI3K), leading to the induction of VEGF and MMP-9 (32, 33). Therefore, we asked whether CD147 affects the transcription of collagen XVIII, which is then cleaved into endostatin. To this end, we extracted total RNA from HT1080 and U937 cells that were separated in co-culture by the inserts and used quantitative PCR (qPCR) to examine the expression levels of collagen XVIIIA messenger RNA (mRNA) in co-culture compared to those in single culture. However, we observed no change in both cell types (**Figures 4A, B**), suggesting that endostatin levels are not regulated by the transcription of its precursor. To strengthen this point, we used Western blot analysis to compare the levels of collagen XVIIIA protein deposited in the ECM by the single-cell culture or co-culture; however, we detected no difference (**Figures 4C, D**). Therefore, we concluded that the regulation of endostatin accumulation occurs posttranscriptionally and posttranslationally, probably at the level of protease cleavage.

### 3.5 Endostatin is cleaved by the specific proteases MMP-9 and proteasome 20S, and CD147 oppositely regulates their activities

Endostatin is a protein derived from the proteolytic cleavage of Col18A by different proteases, including MMP-7, MMP-14, MMP-9, cathepsin S, cathepsin L, elastase, and proteasome 20S. As the expression levels of Col18A were unchanged in co-culture both at the mRNA and at protein levels, we assumed that CD147 affects proteases that cleave endostatin. We, therefore, asked which proteases cleave Col18A in our *in vitro* system. To this end, we used different protease inhibitors and added them to the HT1080 single culture or to the co-culture, in order to identify which protease is relevant to the cleavage of Col18A in our system. The different inhibitors used, their concentrations, and the proteases they inhibit are listed in **Supplementary Table S1**.

**Figure 5:**
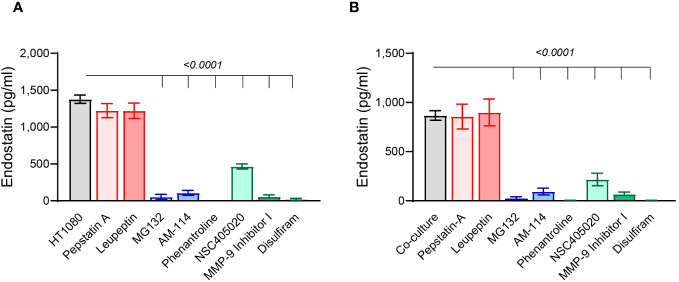
Proteasome 20S and MMP-9 are involved in endostatin generation. The HT1080 cells (3 × 10^4^ cells) in single cultures or in co-culture with U937 cells (3 × 10^4^ cells) were incubated in serum-starvation medium in the presence of TNFα (1 ng/ml), together with different protease inhibitors that were added at the following concentrations: leupeptin 1 μM, pepstatin A 5 μM, MG132 1 μM, AM114 1 μM, phenantroline 40 μM, NSC405020 100 μM, MMP-9 inhibitor I 5 nM, and disulfiram 20 μM. Endostatin levels were evaluated by ELISA in supernatants derived from **(A)** HT1080 single cultures and **(B)** co-cultures (n = 12). Data are presented as means ± SE and were analyzed using one-way ANOVA followed by Dunnett’s *post-hoc* test.

We show that in both HT1080 single cultures and co-cultures with U937 cells, leupeptin and pepstatin A, which inhibit different cathepsins, did not inhibit the generation of endostatin (**Figures 5A, B**), suggesting that in our conditions, cathepsins do not participate in cleaving Col18A. In contrast, both the general inhibitor of MMPs, phenanthroline, and the specific MMP9 inhibitor I inhibited the accumulation of endostatin almost completely. The specific MMP-14 inhibitor NSC405020 inhibited endostatin as well but to a lesser degree. Likewise, the general proteasome inhibitor MG132 and the proteasome 20S–specific inhibitor AM114 also inhibited the accumulation of endostatin almost completely (**Figures 5A, B**). Thus, we concluded that proteasome 20S, MMP-9, and to a lesser extent MMP-14 are implicated in the cleavage of Col18A to endostatin.

**Figure 6:**
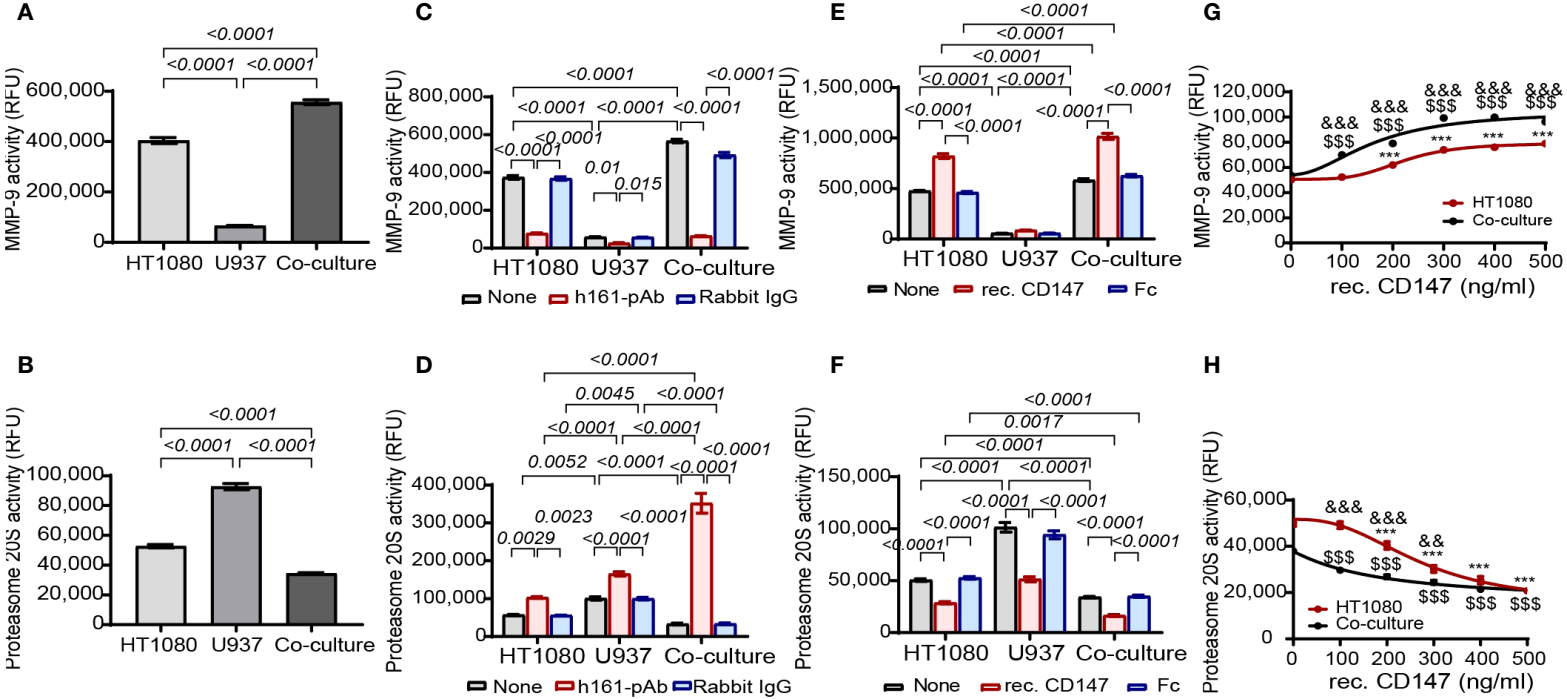
CD147 promotes MMP-9 activity and inhibits proteasome 20S activity. The activities of **(A)** MMP-9 and **(B)** proteasome 20S were evaluated in supernatants derived from HT1080 and U937 single cultures (3 × 10^4^ cells each) or their co-cultures. Data are presented as means ± SE and were analyzed using one-way ANOVA followed by Bonferroni’s *post-hoc* test. **(C, D)** The effect of the addition of h161-pAb or its negative control rabbit IgG (2 ng/ml each) to single cultures and co-cultures or **(E, F)** the addition of recombinant CD147 and its negative control Fc fragment (300 ng/ml each) on **(C, E)** MMP-9 activity and **(D, F)** proteasome 20S activity. The effect of the addition of increasing concentrations of recombinant CD147 on **(G)** MMP-9 activity and **(H)** proteasome 20S activity. Data are presented as means ± SE and were analyzed using two-way ANOVA followed by Bonferroni’s *post-hoc* test. ***, p<0.001 relative to HT1080 cells; $$$, p<0.001 relative to untreated co-culture; &&, p<0.01, &&&, p<0.001 relative to co-culture at the same concentration of rec. CD147.

Focusing on MMP-9 and proteasome 20S, we next asked whether CD147 regulates their activities. To quantify their proteolytic activities, we used fluorescent peptides that are specifically cleaved by either MMP-9 or proteasome 20S. Of note, these peptides do not contain the Col18A sequence; therefore, the release of fluorescence measures only the general activity of these proteases and not the specific activity of Col18A cleavage. First, we show that relative to the single culture, the co-culture enhanced MMP-9 activity and reduced the activity of proteasome 20S (**Figures 6A, B**). The addition of the neutralizing rabbit anti-human CD147 antibody h161-pAb decreased MMP-9 activity in HT1080 cells as well as in the co-culture (**Figure 6C**), but surprisingly, the antibody increased the activity of proteasome 20S in the single cultures and even more so in the co-cultures (**Figure 6D**). This opposite effect of CD147 was further demonstrated by the addition of recombinant CD147 (at a concentration of 300 ng/ml), where MMP-9 activity was enhanced (**Figure 6E**) and the proteasome 20S activity was inhibited (**Figure 6F**). Increasing concentrations of recombinant CD147 also exhibited opposite responses, where MMP-9 activity was gradually increased and the proteasome 20S activity was gradually decreased (**Figures 6G, H**).

TIMP-1 is a known inhibitor of MMP-9, and since we already demonstrated that it is enhanced in the co-culture (**Figure 1F**) and that CD147 inhibits it (**Figure 3D**), we asked whether TIMP-1 participates in the regulation of endostatin generation. To directly assess the effects of TIMP-1, we added increasing concentrations of recombinant TIMP-1 to HT1080 single or co-cultures. The increasing concentrations of recombinant TIMP-1 reduced both endostatin levels and MMP-9 secretion (**Supplementary Figures S2A, B**). Moreover, TIMP-1 inhibited MMP-9 activity (**Supplementary Figure S2C**) but had no effect on the activity of proteasome 20S activity (**Supplementary Figure S2D**).

### 3.6 Proteasome 20S and MMP-9 synergize, and CD147 regulates their activity in a hormetic manner, outside the cells

We have demonstrated that MMP-9 inhibitor I or AM114, the specific inhibitors of MMP-9 and proteasome 20S, respectively, can each independently inhibit almost completely the cleavage of endostatin from Col18A (**Figure 5**). However, the fact that the inhibition of only one protease leads to almost negligible concentration of endostatin suggests that the two proteases affect each other, as the non-inhibited protease is unable to generate enough endostatin on its own. Additionally, since Col18A is deposited outside the cells and CD147, proteasome 20S, and MMP-9 are all secreted to the supernatants, it is likely that the cleavage of Col18A is carried out outside the cells.

Accordingly, we have changed our experimental system to a non-cellular system. As commercial Col18A is unavailable and because we know it exists in our system as endostatin is generated, we now incubated HT1080 cells alone for 72 h, where levels of endostatin were the highest, and allowed them to deposit their ECM proteins. Next, we destroyed the cells with a hypotonic solution (DDW) and washed away cell debris. The resulting ECM contained Col18A, as seen in the Western blots (**Figure 4C**). Proof that this new non-cellular system works was obtained first by evaluating the levels of endostatin in the ECM that was untreated, in the supernatants derived from HT1080 single cultures and in the conditioned media where ECM was incubated with the same supernatants (**Supplementary Figure S3A**). When ECM was incubated with the HT1080-derived supernatants, the levels of endostatin were significantly elevated compared to the levels of endostatin in these supernatants prior to their incubation with the ECM. Also, the concentrations of endostatin and the activities of MMP-9 and proteasome 20S (**Supplementary Figures S3B–D**) presented with the same trends observed before in the cellular system when the h161-pAb antibody was introduced (**Figures 2A**, **6C, D**).

To be able to observe possible interactions between MMP-9 and proteasome 20S, we needed to work in lower concentrations of inhibitors and enzymes. Therefore, we first calibrated the concentrations of proteasome 20S and recombinant MMP-9 that yielded approximately 20% of the respective maximal activity of endostatin generation in the non-cellular system (**Supplementary Figures S4A, B**). For proteasome 20S, it was 0.15 nM, and for recombinant MMP-9, the chosen concentration was 0.2 ng/ml. Similarly, the concentrations of the inhibitors were determined by their ability to inhibit only approximately 25% of the ability to generate endostatin in this system (**Supplementary Figures S4C, D**). For the proteasome 20S inhibitor AM114, the concentration chosen was 1 nM, and for MMP-9 inhibitor I, the concentration chosen was 0.05 nM.

**Figure 7:**
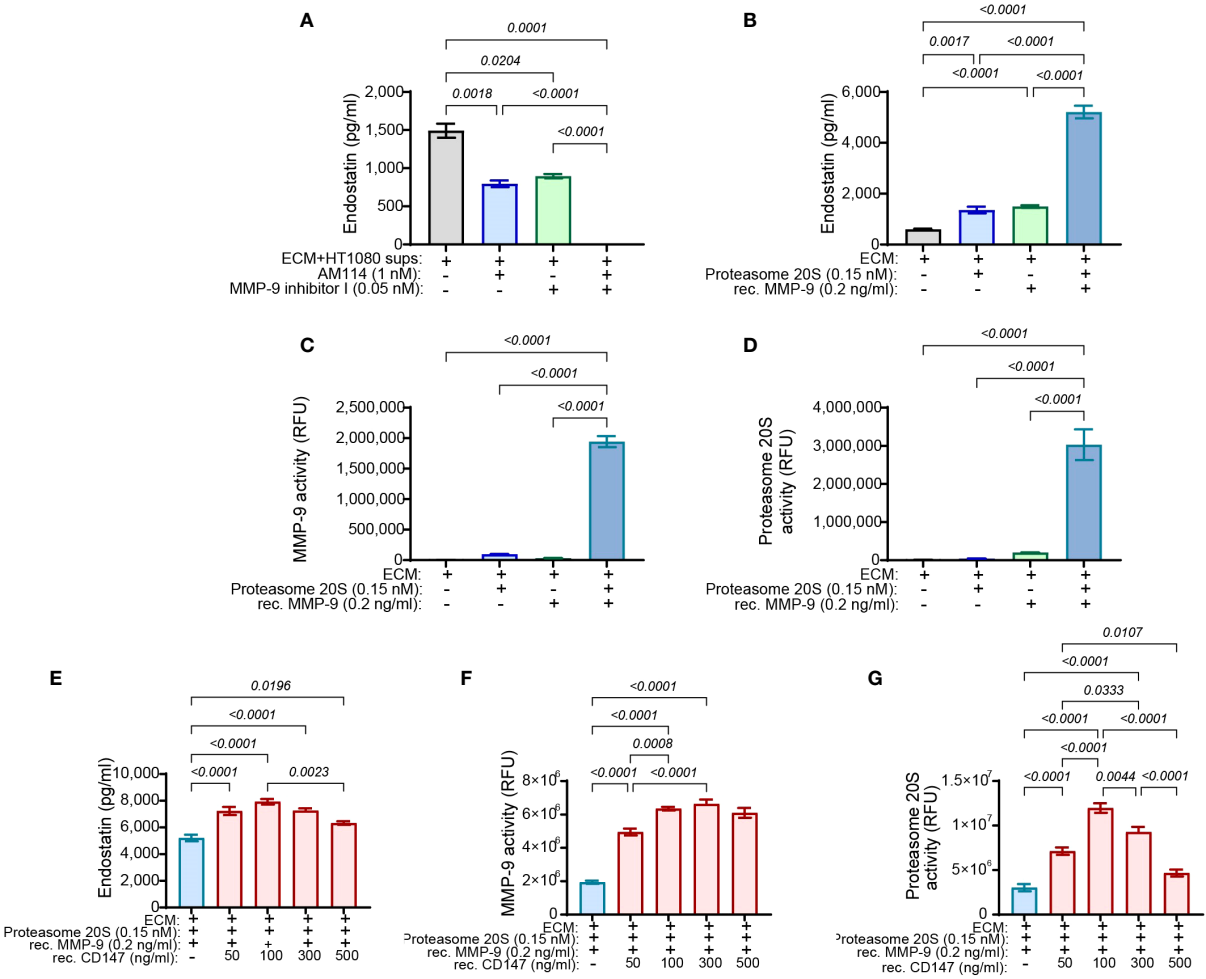
MMP-9 and proteasome 20S synergistically enhance the cleavage of endostatin from Col18A, and CD147 regulates endostatin and the activity of proteasome 20S in a hormetic manner. HT1080 (3 × 10^4^ cells) were incubated in serum-starvation medium with TNFα (1 ng/ml) for 72 h and allowed to deposit their ECM proteins. Cells were destroyed with DDW for 20 min, and cellular debris was washed away with PBS three times. The ECM was then incubated with the indicated concentrations of **(A)** HT1080-derived supernatants together with AM114 and MMP-9 inhibitor I (n = 6) or with **(B)** proteasome 20S that was activated with 0.035% SDS and recombinant MMP-9 that was activated by incubation with APMA (1 mM) (n = 12). The activity of **(C)** MMP-9 (n = 6) or **(D)** proteasome 20S (n = 6) was measured as well. The generation of endostatin and the activities of MMP-9 and proteasome 20S were synergistically enhanced in the presence of both enzymes. Additionally, HT1080-derived ECM was incubated with recombinant MMP-9 (0.2 ng/ml) and proteasome 20S (0.15 nM) and with increasing concentrations of recombinant CD147 for 48 h. Supernatants were collected, and **(E)** the levels of endostatin, **(F)** the activity of proteasome 20S, and **(G)** the activity of MMP-9 were evaluated (n = 6). Data are presented as means ± SE and were analyzed using one-way ANOVA followed by Bonferroni’s *post-hoc* test.

To demonstrate that MMP-9 and proteasome 20S work in synergy, the supernatants derived from the HT1080 culture were incubated together with the chosen concentrations of MMP-9 inhibitor I and AM114. After 48 h of incubation, the levels of the endostatin measured in the supernatants demonstrated that while each inhibitor could reduce the levels of endostatin, their combination completely inhibited endostatin formation (**Figure 7A**). This was also reflected in the functional wound assay, where these treatments resulted in the increased angiogenic potential (**Supplementary Figure S6A**). In the complementary experiment, the ECM was incubated for 48 h with the recombinant MMP-9 and proteasome 20S in the chosen concentrations and the accumulation of endostatin was measured. Each of the enzymes generated some endostatin, but their combination synergistically increased endostatin levels (**Figure 7B**). Again, in the wound assay, increased levels of endostatin resulted in reduced migration of endothelial cells (**Supplementary Figure S6B**). The general activity of the two enzymes in the supernatants was evaluated and showed that the combination of the enzymes synergistically enhanced both of their activities (**Figures 7C, D**). These results suggest that proteasome 20S promotes the activity of MMP-9, and MMP-9 promotes the activity of proteasome 20S.

As we already observed that CD147 regulates endostatin and oppositely regulates proteasome 20S and MMP-9, we now asked whether it could directly affect their activities and the generation of endostatin in the non-cellular system. To this end, we used the ECM in the non-cellular system and added the chosen concentrations of MMP-9 and proteasome 20S, each alone or together, and we observed the same synergistic effect as before (blue bar, **Figure 7E**). Increasing concentrations of recombinant CD147 were now added in addition to the presence of proteasome 20S and recombinant MMP-9. Surprisingly, CD147 affected the accumulation of endostatin in a hormetic manner, peaking at 100 ng/ml (**Figure 7E**). Moreover, the activity of proteasome 20S was also affected in a hormetic manner, reaching a peak at 100 ng/ml (**Figure 7G**), whereas MMP-9 was not significantly affected by the addition of increasing concentrations of CD147 (**Figure 7F**).

The levels of endostatin measured in our experiments are the steady-state levels, which reflect the balance between the cleavage of endostatin from Col18A by proteasome 20 and MMP-9 and its degradation by other, yet unidentified, proteases. Thus, it is possible that a different rate of degradation between single HT1080 cultures and co-cultures with U937 cells accounts for the reduced levels observed in co-cultures (**Figure 1D**). To examine this possibility, we prepared ECM as before, and incubated it with supernatants derived from the HT1080 single culture or from the co-cultures for 24 h to allow endostatin to be generated. Then, MMP-9 inhibitor I and the proteasome 20S inhibitor AM114 were added to stop the generation of new endostatin molecules, and samples from the supernatants were taken over time to determine the amounts of endostatin. We show that the rate of degradation in the HT1080 single culture or in the co-cultures was similar, and the half-life of the endostatin protein was estimated at approximately 24 h (**Supplementary Figure S5**). Thus, degradation was ruled out as a possible explanation for the difference in endostatin levels between HT1080 single culture and their co-culture with U937 monocytes.

### 3.7 CD147 directly regulates MMP-9 and proteasome 20S activities, suggesting that it acts as an allosteric factor

The ability of CD147 to regulate the activities of both MMP-9 and proteasome 20S suggests that the three proteins collaborate to generate endostatin from Col18A. However, even in our non-cellular ECM system, the ability to generate endostatin depends on the amount of Col18A deposited in the ECM and on the ability of other proteins that may be present in the ECM, yet undefined, to affect the proteases’ activities and products.

**Figure 8:**
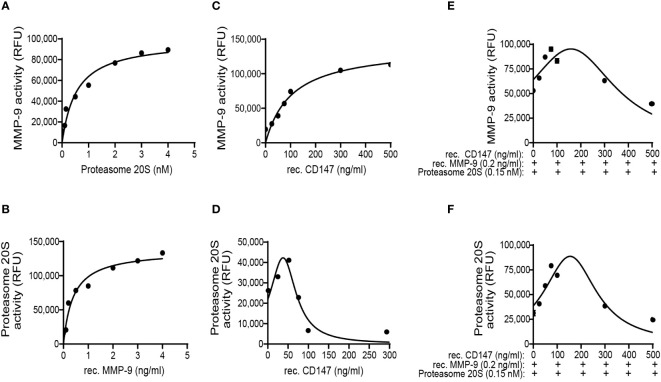
MMP-9 and proteasome 20S promote each other’s activities, and CD147 hormetically regulates proteasome 20S activity or the combination of the two proteases. The effects of the three proteins recombinant (rec.) MMP-9, proteasome 20S, and rec. CD147 on each other’s activity were investigated in an isolated, non-cellular system and in the absence of any other proteins. **(A)** Increasing concentrations of proteasome 20S were added to a constant concentration of MMP-9 (0.2 ng/ml), and MMP-9 activity was measured (n = 6). **(B)** Increasing concentrations of MMP-9 were added to a constant concentration of proteasome 20S (0.15 nM), and proteasome 20S activity was measured (n = 6). Increasing concentrations of rec. CD147 were added to **(C)** a constant concentration of MMP-9 (0.2 ng/ml, n = 6) or **(D)** a constant concentration of proteasome 20S (0.15 nM, n = 6). Increasing concentrations of rec. CD147 were added to a mixture of a constant concentration of MMP-9 (0.2 ng/ml) and a constant concentration of proteasome 20S (0.15 nM), and the activity of **(E)** MMP-9 or **(F)** proteasome 20S was measured (n = 6).

To rule out the second possibility and isolate any interference, we now added two or three of the components in a tube, in the absence of ECM. When MMP-9 was held at a constant concentration (0.2 ng/ml) and increasing amounts of proteasome 20S were added to the tube, MMP-9 activity was increased, demonstrating that proteasome 20S can enhance MMP-9 activity (**Figure 8A**). When proteasome 20S was held at a constant concentration (0.15 nM), increasing amounts of MMP-9 enhanced its activity (**Figure 8B**). Similar to our previous findings in the ECM system, when MMP-9 was held at a constant concentration (0.2 ng/ml) and CD147 was added in increasing amounts, the activity of MMP-9 increased (**Figure 8C**). More interestingly, addition of increasing amounts of CD147 to a constant concentration of proteasome 20S (0.15 nM) resulted in a hormetic response, where proteasome 20S activity was initially increased, peaking at 50 ng/ml of CD147 (**Figure 8D**), before decreasing again. When all three components were added in the tube, keeping proteasome 20S and MMP-9 at their constant concentrations and adding increasing amounts of CD147, a hormetic response was observed (**Figures 8E, F**). These results suggest that CD147 hormetically regulates proteasome 20S, but not MMP-9 activities. Because MMP-9 and proteasome 20S affect each other, the hormetic effect of CD147 on proteasome 20S could also affect MMP-9 activity.

### 3.8 Angiogenesis is enhanced in RA patients via CD147

**Figure 9:**
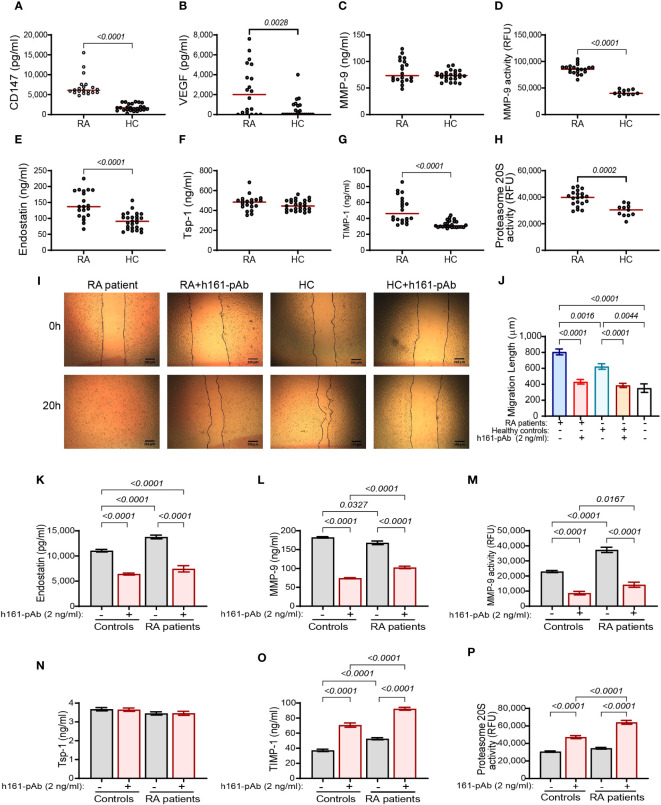
The effect of disease on the serum levels of RA patients. Serum samples from patients with active RA were collected (RA, n = 23) and the concentrations of **(A–C, E–G)** the pro- and anti-angiogenic factors were compared to those in the serum of healthy volunteers (HC, n = 25). **(D, H)** Additionally, the activities of proteasome 20S and MMP-9 were evaluated in the serum samples. Data are presented as median values (red) and were analyzed using the non-parametric Mann–Whitney test. **(I, J)** The functional wound assay was carried out using cells of the confluent human endothelial cell line EaHy926 that were scratched. Serum samples from RA patients (n = 12) or controls (n = 12) were diluted 1:2 in full medium and applied onto the endothelial layer, with or without the presence of the rabbit anti-human CD147 antibody (h161-pAb, 2 ng/ml). Images were taken at the beginning of the experiment (0 h) and after 20 h. The distance between the two sides of the scratch at 20 h was subtracted from the distance at 0 h, to calculate the net distance that endothelial cells migrated to. Data are presented as means ± SE and were analyzed using the one-way ANOVA test followed by Bonferroni’s *post-hoc* test. **(K–P)** The human HT1080 fibroblast cell line (3 × 10^4^ cells) was seeded in 96-well plates and incubated in serum-starvation medium for 48 h in the presence of TNFα (1 ng/ml) and the serum samples (diluted 1:2) obtained from controls or RA patients and with or without h161-pAb (2 ng/ml). Then, supernatants were collected, and the concentrations of **(K)** endostatin (n = 13), **(L)** MMP-9 (n = 13), **(N)** Tsp-1 (n = 13), and **(O)** TIMP-1 (n = 13) were determined by ELISA. The activities of **(M)** MMP-9 (n = 13) and **(P)** proteasome 20S (n = 13) were measured. Data are presented as means ± SE and were analyzed using the one-way ANOVA test followed by Bonferroni’s *post-hoc* test.

To show whether the findings in the *in vitro* system are also relevant *in vivo*, the concentrations of both pro-angiogenic factors and anti-angiogenic factors in the serum of RA patients that were treated with methotrexate (MTX) or other conventional disease-modifying antirheumatic drugs (cDMARDs) in comparison to those in healthy volunteers as controls were evaluated. Both the levels of endostatin and CD147 are elevated in the serum of RA patients (**Figures 9A, E**), as are the levels of VEGF and TIMP-1 (**Figures 9B, G**). In contrast, the levels of Tsp-1 and MMP-9 are comparable (**Figures 9C, F**). Moreover, both the activities of MMP-9 and proteasome 20S are elevated in the RA patients (**Figures 9D, H**). These data are only partially in agreement with the *in vitro* data that show elevated levels of CD147, MMP-9, VEGF, and TIMP-1 in the co-culture but decreased levels of endostatin and Tsp-1, whereas in the serum of RA patients, the levels of endostatin are increased. This discrepancy could be explained by the different natures of the experimental conditions. The serum presents the sum of interactions between many cell types in many organs and tissues and represents in fact a multicellular co-culture. Many cell types can interact with the fibroblasts in the diseased joint, in addition to monocytes (e.g., T cells, B cells, and endothelial cells), and those interactions could affect the overall production of endostatin. Additionally, in contrast to the *in vitro* setting where only TNFα was added, in RA patients, other pro-inflammatory cytokines are elevated locally in the joints and systemically in the serum (e.g., IL-1β, IL-6, and IL-17, and their effects on the proteases, CD147, and endostatin are not represented in the *in vitro* system. Therefore, it is possible that such factors are involved in the *in vivo* regulation of both CD147 and endostatin, and their effect should be identified and further investigated. To rule out the possibility that MTX or other cDMARDs affect the results, we compared our MTX-treated RA cohort to a previous cohort that included eight patients with active RA disease that were not treated with MTX, other cDMARDs, or high-dose steroids (≤5 mg daily). We show that the levels of CD147, endostatin, and proteasome 20S activity are not different between the two RA patient cohorts (**Supplementary Figures S7A–C**), whereas MMP-9 activity was reduced by cDMARDs (**Supplementary Figure S7D**), in accordance with their known ability to inhibit pro-inflammatory mediators (34).

To explore whether the disease affects the angiogenic potential of the patients and whether CD147 has a regulatory role here as well, we performed the wound assay and added the patients’ serum samples to a confluent layer of EaHy926 endothelial cells that had been previously scratched. The rate of the endothelial cell migration to close the gap (wound) generated by the scratch was measured, with or without the presence of the rabbit anti-human CD147 antibody (h161-pAb, 2 ng/ml). The disease increased the angiogenic potential relative to that of the control samples, and the neutralizing anti-CD147 antibody inhibited this effect, indicating that CD147 regulates angiogenesis *in vivo* as well (**Figures 9I, J**).

Lastly, to show that CD147 also regulates the generation of endostatin *in vivo*, we added the diluted serum samples from RA patients or their healthy controls, with or without the presence of the neutralizing rabbit anti-human CD147 antibody (h161-pAb, 2 ng/ml), to the single HT1080 culture. Endostatin and MMP-9 activities, but not MMP-9 expression, were increased in RA patients, and h161-pAb reduced them all in controls and in RA patients (**Figures 9K–M**). While Tsp-1 remained comparable to controls, TIMP-1 levels were increased in RA patients, and h161-pAb increased them further (**Figures 9N, O**). The proteasome 20S activity was not changed in RA patients relative to that in the controls, but the h161-pAb increased the activity of proteasome 20S in both groups (**Figure 9P**). Thus, the interaction between CD147, endostatin, MMP-9, and proteasome 20S was verified in RA patients.

## 4 Discussion

In this study, we show for the first time that CD147 affects the activity of MMP-9 and secreted proteasome 20S to hormetically regulate the generation of the anti-angiogenic factor endostatin outside the cells. We and others have previously shown that CD147 induces MMP-9 and VEGF and promotes angiogenesis, even in its secreted form (21, 35). Furthermore, interactions between fibroblasts and monocytes in the synovium, which are partly mediated by CD147, were shown to drive angiogenesis and promote RA disease progression (1, 20, 21). However, now we provide evidence that suggests that the role of CD147 is more complex and that it affects not only pro-angiogenic factors but also the anti-angiogenic factor endostatin, thereby suggesting a key function for CD147 in the angiogenic switch.

In our experiments, we show an increase in VEGF, MMP-9, and TIMP-1 in the co-cultures relative to their levels in the single HT1080 cultures and a decrease in the anti-angiogenic factors endostatin and Tsp-1 (**Figure 1**). However, by neutralizing CD147 with h161-pAb, knocking down its expression, or adding exogenous CD147, we demonstrate that CD147 specifically regulates endostatin, but not Tsp-1 levels (**Figure 2**; **Supplementary Figure S1**). This seems contradictory, as it suggests that CD147 can simultaneously turn the angiogenic switch both on and off. However, the hormetic response to CD147, peaking at 250–300 ng/ml, suggests that the regulation of endostatin generation depends on the ambient concentrations of CD147 (**Figure 3C**).

**Figure 10:**
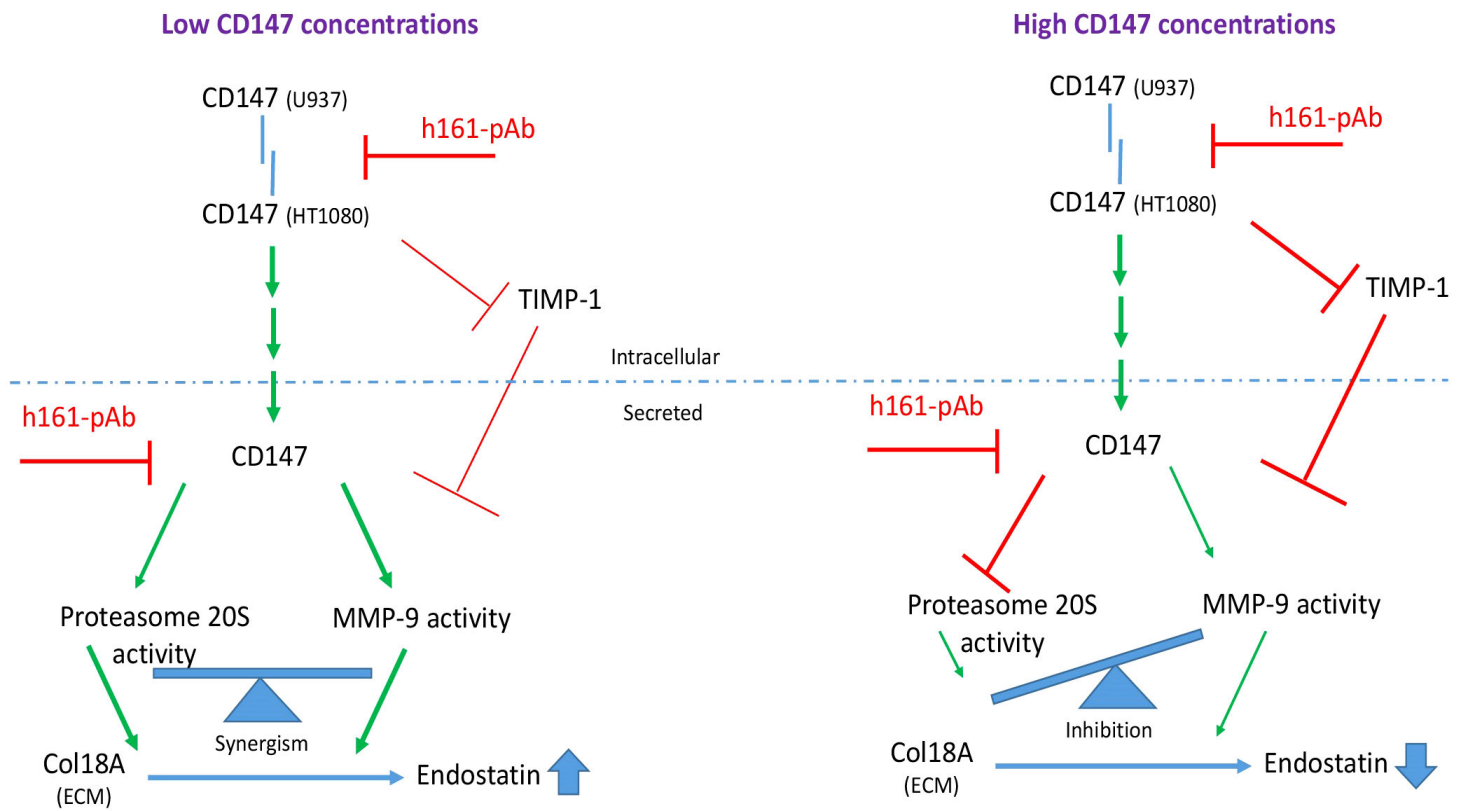
Schematic model of the regulatory role of soluble CD147 in the generation of endostatin. When CD147 is secreted in low amounts, it enhances the activities of both MMP-9 and secreted proteasome 20S. These two enzymes collaborate and synergize to cleave Col18A and generate endostatin. When CD147 is secreted in high concentrations, it inhibits TIMP-1, which in turn inhibits MMP-9 activity, and it inhibits proteasome 20S activity. Because proteasome 20S and MMP-9 must collaborate to generate endostatin, the strong inhibition of only one of them, proteasome 20S in this case, is sufficient to inhibit endostatin generation.

One way to explain the hormetic effect of CD147 on the generation of endostatin is its hormetic and inhibitory effect on TIMP-1 (**Figure 3D**), the selective inhibitor of MMP-9 (9), which we describe here. This finding is contradictory to previous findings that show induction of TIMP-1 by CD147 (36). However, this discrepancy might be explained by the hormetic response to different concentrations of CD147. Of note, hormetic responses are shared by other anti-angiogenic agents (37, 38), and we have demonstrated the hormetic effects of CD147 or its neutralizing antibody in other systems (39, 40). Generally, TIMP-1 is considered an anti-angiogenic factor, because of its ability to inhibit MMPs, MMP-9 in particular. However, previous studies have suggested that TIMP-1 may also have pro-angiogenic activities (41, 42). We suggest that TIMP-1 activities are complex, as by inhibiting the pro-angiogenic factor MMP-9, it could also inhibit the anti-angiogenic factor endostatin. Moreover, CD147 may indirectly be responsible for this fine balance (**Figure 10**) and act as a regulatory switch that can turn the angiogenic switch on or off, according to its local concentrations.

CD147 is known to transduce signaling pathways such as those of PI3K and MAPKs (43), suggesting that it could regulate the transcription or translation of Col18A. However, the expression of Col18A mRNA and protein levels in co-culture was unchanged relative to that in the single cultures (**Figure 4**). Thus, we concluded that the regulation of endostatin generation must occur at the level of proteolytic cleavage. The 22-kDa endostatin is the cleavage product of the C-terminal fragment of Col18A, and several proteases, including cathepsins S and L, MMP-7, MMP-9, and MMP-14, and even proteasome 20S have been implicated in its cleavage (25, 28–30). Using protease inhibitors, we demonstrate that in our *in vitro* system, MMP-9 and proteasome 20S are responsible for the cleavage of endostatin from Col18A, whereas the cathepsin inhibitors had no impact (**Figure 5**). Moreover, we demonstrate that CD147 oppositely regulates the activities of these two proteases, as the neutralizing h161-pAb antibody inhibits MMP-9 activity and promotes proteasome 20S activity, and recombinant CD147 enhances MMP-9 activity and inhibits proteasome 20S activity (**Figure 6**).

As Col18A is deposited outside the cells and MMP-9, proteasome 20S, and CD147 are all secreted proteins, we next predicted that the cleavage of Col18A into endostatin also occurs outside the cells. Setting up the non-cellular system allowed us to distinguish between the activities of CD147 as a ligand that binds to its receptor in a homophilic interaction that triggers a signaling pathway, and CD147 that acts directly on the proteins outside the cells. First, we demonstrate that MMP-9 and proteasome 20S are both required for the cleavage of endostatin and that their cooperation synergistically enhances the proteolytic cleavage of Col18A and generates endostatin (**Figure 7**). Thus, when one protease is inhibited, the other cannot compensate for it and endostatin is reduced or completely abolished (**Figure 5**). Moreover, the addition of one protease in a constant limiting concentration and the other protease in increasing concentrations, where no other proteins were included in the assay, demonstrated this cooperation and suggested a positive allosteric regulation (**Figures 8A, B**).

While MMP-9 is a protease known to be secreted, proteasome 20S is mostly considered a cytoplasmic core component of the larger proteasome 26S machinery, dedicated to the proteolytic degradation of damaged or misfolded proteins within the cell (44). However, there is now increasing recognition of the regulated presence of extracellular proteasome 20S in different body fluids, including synovial fluids and plasma (45, 46). The manner in which proteasome 20S arrives at these locations and the biological roles it plays there are only now beginning to unfold. Specifically, only one study we are aware of implicated proteasome 20S in the cleavage of Col18A to generate endostatin (30). An interplay between proteasome and MMPs has been previously described. For example, inhibition of the proteasome with MG132 inhibited the myocardial expression of MMP-2 and MMP-9 (47). The proteasome inhibitor bortezomib in combination with the histone deacetylase inhibitor romidepsin inhibited the expression of MMP-2 and MMP-9 in A549 non-small-cell lung cancer (NSCLC) cells (48). The proteasome irreversible inhibitor epoxomicin enhanced the mRNA expression of MMP-2 and reduced the mRNA expression of TIMP-1 in the human retinal pigment epithelial cells ARPE-19 (49). However, these examples all refer to the regulation of expression of the MMPs by the proteasome and not to the regulation of their proteolytic activity. We are unaware of any study that demonstrated a direct regulation on the activity of either proteasome or any MMP, exerted by these proteases on each other. To the best of our knowledge, this is the first time that a mutual, bidirectional, possibly allosteric regulation of the proteolytic activities of proteasome 20S and MMP-9 is demonstrated.

In this study, we show that recombinant CD147 that was exogenously added to the non-cellular ECM system, in the presence of limiting concentrations of MMP-9 and proteasome 20S, hormetically enhanced endostatin production (**Figures 7E–G**). Soluble CD147 molecules were previously known to bind to the transmembranal CD147 that acted as a receptor to trigger signaling. However, here we demonstrate that in the absence of any cells, the soluble CD147 molecule can act independently, not relying on the presence of a receptor CD147 molecule. This hormetic, bell-shaped response was best exemplified on the activity of proteasome 20S (**Figures 7G, D**). Such a hormetic response was observed for MMP-9 activity only when all three components, CD147, proteasome 20S, and MMP-9, were present together in the same non-cellular and non-ECM assay (**Figure 8E**), suggesting that CD147 affects mostly the proteasome 20S activity. Collectively, these results suggest, for the first time, that soluble CD147 has an extracellular role that is independent of the CD147 receptor and does not rely on cell signaling. In fact, the results assign to CD147 a new role as a possible allosteric regulator of proteasome 20S activity and of the generation of endostatin. This ability of CD147 to alter proteasome 20S activity might have implications on other processes, such as proliferation and apoptosis, that were not studied here.

The results that were found using *in vitro* systems were corroborated in serum samples obtained from RA patients. The readily available serum samples were not the first choice for our study, as they reflect the systemic condition of RA patients. However, we had no access to the actual joint of patients and, therefore, could not acquire primary fibroblast-like synoviocytes (FLSs), which could better reflect the condition in the joint. The pro-angiogenic factors CD147, VEGF and TIMP-1, as well as MMP-9 activity, but not its expression, and proteasome 20S activity were elevated in the serum samples of RA patients relative to the controls (**Figures 9A–H**) or when those serum samples were added to the HT1080 fibroblast cell line (**Figures 9K–P**), whereas the levels of Tsp-1 were unchanged from those in controls. The overall angiogenic potential, which was assessed by the wound assay, was elevated in RA patients, and CD147 was important, as the antibody decreased the angiogenic activity to the basal levels in both groups (**Figures 9I, J**). These data are generally in agreement with the *in vitro* results, with the exception of the elevation in endostatin levels (**Figure 9E**). This discrepancy could be explained by the use of serum samples, which include a myriad of cytokines secreted by different cell types that are not present in the co-culture system that includes only two cell types, and might have affected the levels of endostatin in the serum of the RA patients. The possibility that treatment with cDMARDs, including MTX, was responsible for this discrepancy was ruled out, since our RA patients that were all treated with MTX or other cDMARDs showed no difference in their levels of endostatin, CD147, or proteasome 20S activity compared to those of untreated RA patients with active disease from a different cohort (**Figure S7**). However, cDMARDs did reduce MMP-9 activity, in agreement with their inhibitory effect on pro-inflammatory cytokines (34), emphasizing the dominant role of proteasome 20S over that of MMP-9 in the regulation of endostatin production and pointing out that disease activity affects the angiogenic potential. It seems that in the serum samples obtained from RA patients, CD147 levels are in the low range, relative to the *in vitro* levels, allowing the increase in endostatin levels, as described in the model (**Figure 10**).

Taking into account all of the results, we propose the following model that distinguishes between low and high CD147 concentrations (**Figure 10**). CD147 can act as a receptor and upon fibroblast–monocyte interactions trigger the signaling pathway that induces MMP-9 expression. CD147 can also gradually inhibit TIMP-1 to enhance the activity of MMP-9. Additionally, CD147, MMP-9, and proteasome 20S are secreted from the cells, where this trio of proteins can cooperate to enhance the activities of MMP-9 and proteasome 20S and specifically to enhance endostatin generation. In contrast, when the concentrations of soluble CD147 are higher, proteasome 20S activity is strongly inhibited, leading to decreased generation of endostatin.

## Data availability statement

The raw data supporting the conclusions of this article will be made available by the authors, without undue reservation.

## Ethics statement

The studies involving humans were approved by Carmel Medical Center IRB, approval number CMC-0130-17; Bnai Zion Medical Center IRB, approval number 0142-17-BNZ. The studies were conducted in accordance with the local legislation and institutional requirements. The participants provided their written informed consent to participate in this study.

## Author contributions

MMR: Investigation, Methodology, Writing – original draft. HS: Investigation, Methodology, Writing – original draft. ES: Investigation, Methodology, Writing – original draft. AH: Resources, Writing – original draft. TG: Resources, Writing – original draft. JF: Resources, Writing – original draft. GS: Resources, Writing – original draft. AK: Resources, Writing – original draft. ME: Resources, Writing – original draft. DZ: Funding acquisition, Writing – review & editing. MAR: Conceptualization, Formal Analysis, Supervision, Writing – original draft, Writing – review & editing.
