# Supplementary material for: Soluble CD147 regulates endostatin via its effects on the activities of MMP-9 and secreted proteasome 20S
Source: Front Immunol. 2024 Jan 22;15:1319939. doi: 10.3389/fimmu.2024.1319939 (PMC10840997; doi:10.3389/fimmu.2024.1319939)
Supplement: Supplementary file 1 [file DataSheet_1.docx]

# **Supplementary Data**

# **Table S1: Protease inhibitors used in the study**

| **Company** | **Concentrations Used** | **Target/Specificity** | **Inhibitor** |
| --- | --- | --- | --- |
| Merck | 1 μg | Serine/cysteine/threonine proteases (e.g., cathepsin D, E) | Leupeptin |
| Merck | 5 μM | Aspartic acid proteases  (e.g., cathepsin S, L, D, K) | Pepstatin A |
| Merck | 40 μM | MMPs - wide range | Phenanthroline |
| R&D systems | 100 μM | Specific for MMP-14 | NSC 405020 |
| Cayman | 5 nM | Specific for MMP-9 | MMP-9 Inhibitor I |
| R&D systems | 20 μM | Specific for both MMP-9 and Proteasome 20S | Disulfiram |
| Merck | 1 μM | General for proteasome | MG-132 |
| R&D systems | 1 μM | Specific for proteasome 20S | AM 114 |

# **Table S2: Primers used for qPCR analysis**

| **Gene** | **Fw sequence** | **Rv sequence** |
| --- | --- | --- |
| Col18A | 5'-CACCACAGCTAGGTGCA | 5'-CGCAACCAGGTGGAGCAC |
| PBGD | 5'-CAGTTTGAAATCATTGCTAT | 5'-CTCCAATCTTAGAGAGTGCA |

**
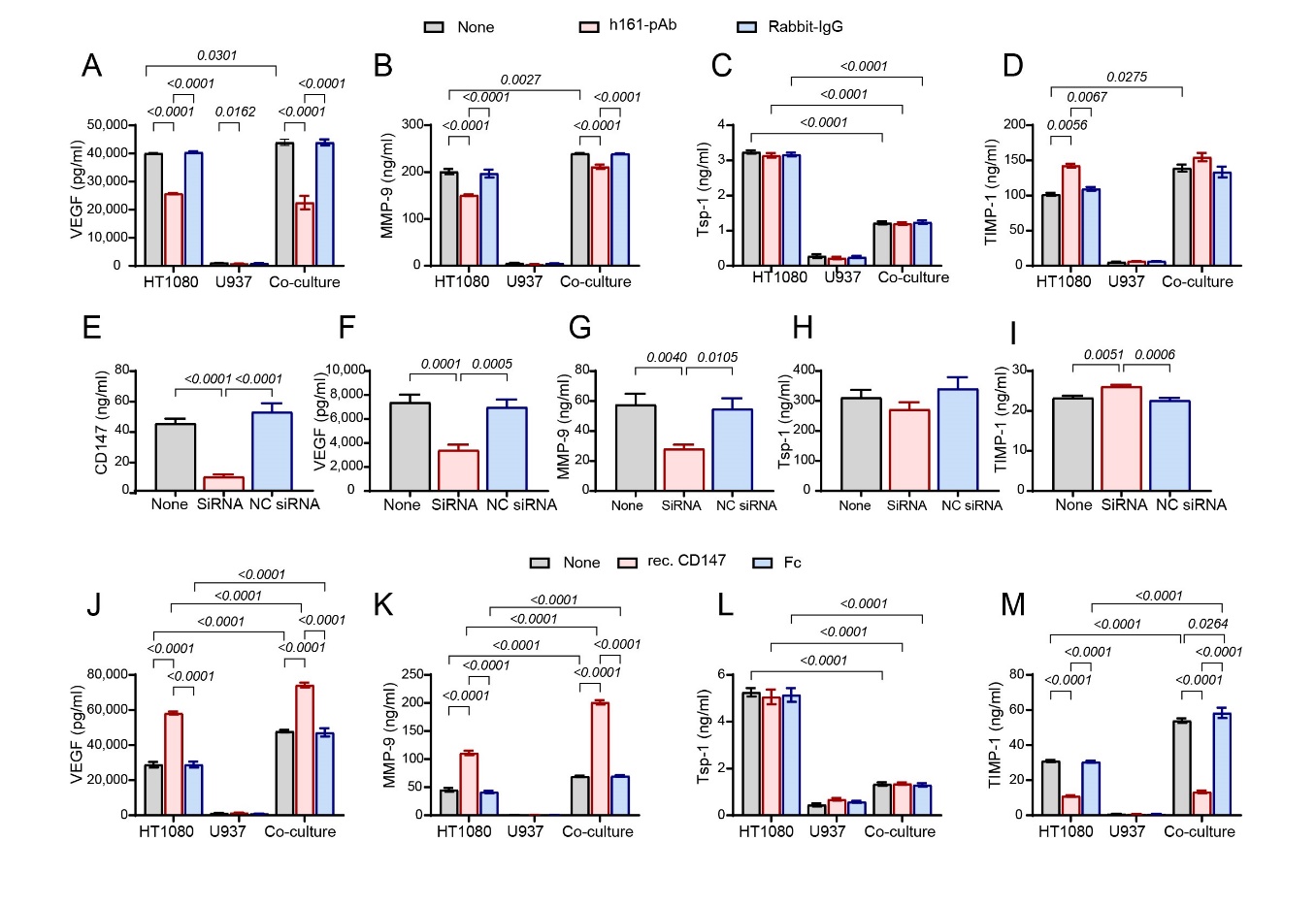
**

**Figure S1: CD147 induces the pro-angiogenic factors VEGF, MMP-9 and the anti-angiogenic factor TIMP-1, but not Tsp-1.** HT1080 cells (3x10^4^ cells) were incubated alone or in co-culture with U937 cells (3x10^4^ cells) for 48h in the presence of TNFα (1ng/ml). **(A-D)** the cells were incubated alone, with the anti-CD147 antibody (h161-pAb, 2ng/ml) or with an irrelevant antibody (Rabbit-IgG, 2 ng/ml). After 48h incubation, supernatants were collected, and the concentrations of **(A)** VEGF, **(B)** MMP-9, **(C)** Tsp-1, and **(D)** TIMP-1 were assessed using ELISA (n=6). Data are presented as means ± SE, and were analyzed using two-way ANOVA followed by the Bonferroni's post hoc test**. (E-I)** The human HT1080 fibroblast cell line (10^5^ cells) was transfected with two CD147 siRNA molecules (10 nM each) and left for 24 h in full medium. Then the medium was replaced with serum-starvation medium (antibiotics-free) with TNFα (1ng/ml), and U937 cells (10^5^ cells) were added in inserts (0.4 μm pore size). After 48 h incubation in co-cultures, supernatants were collected, and concentrations of **(E)** CD147 (n=7), **(F)** VEGF (n=7), **(G)** MMP-9 (n=7), **(H)** Tsp-1 (n=6), and **(I)** TIMP-1 (n=6) were assessed using ELISA. **(J-M)** HT1080 (3x10^4^ cells), U937 cells (3x10^4^ cells) and their co-culture were incubated in serum starvation medium with TNFα (1ng/ml), in the presence of the human recombinant CD147 (300 ng/ml) or the Fc fragment (300 ng/ml) for 48h. After incubation, supernatants were collected, and concentrations of **(J)** VEGF (n=6), **(K)** MMP-9 (n=6), **(L)** Tsp-1 (n=6), and **(M)** TIMP-1 (n=6), were assessed by ELISA**.** Data are presented as means ± SE, and were analyzed using two-way ANOVA followed by the Bonferroni's post hoc test.

**
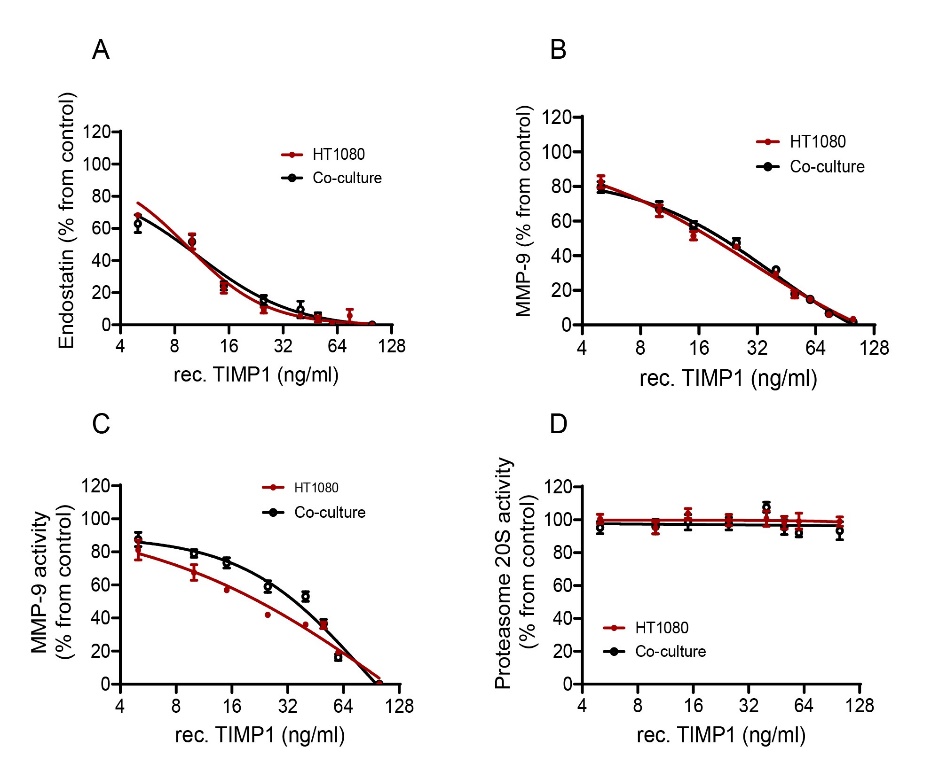
**

**Figure S2: TIMP-1 inhibits MMP-9 secretion and activity, but has no effect on proteasome 20S activity.** HT1080 (3x10^4^ cells), U937 cells (3x10^4^cells) or their co-culture were incubated in serum-starvation medium with TNFα (1ng/ml), and with increasing concentration of rec. TIMP-1 for 48h. Supernatants were collected and the concentrations of **(A)** endostatin (n=6), or **(B)** MMP-9 (n=6), were determined using ELISA, and their percentage from the unstimulated cells is presented. The activities of **(C)** MMP-9 (n=6) and **(D)** proteasome 20S (n=6) were determined using their respective fluorescent peptide substrates. Data are presented as means ± SE.

**
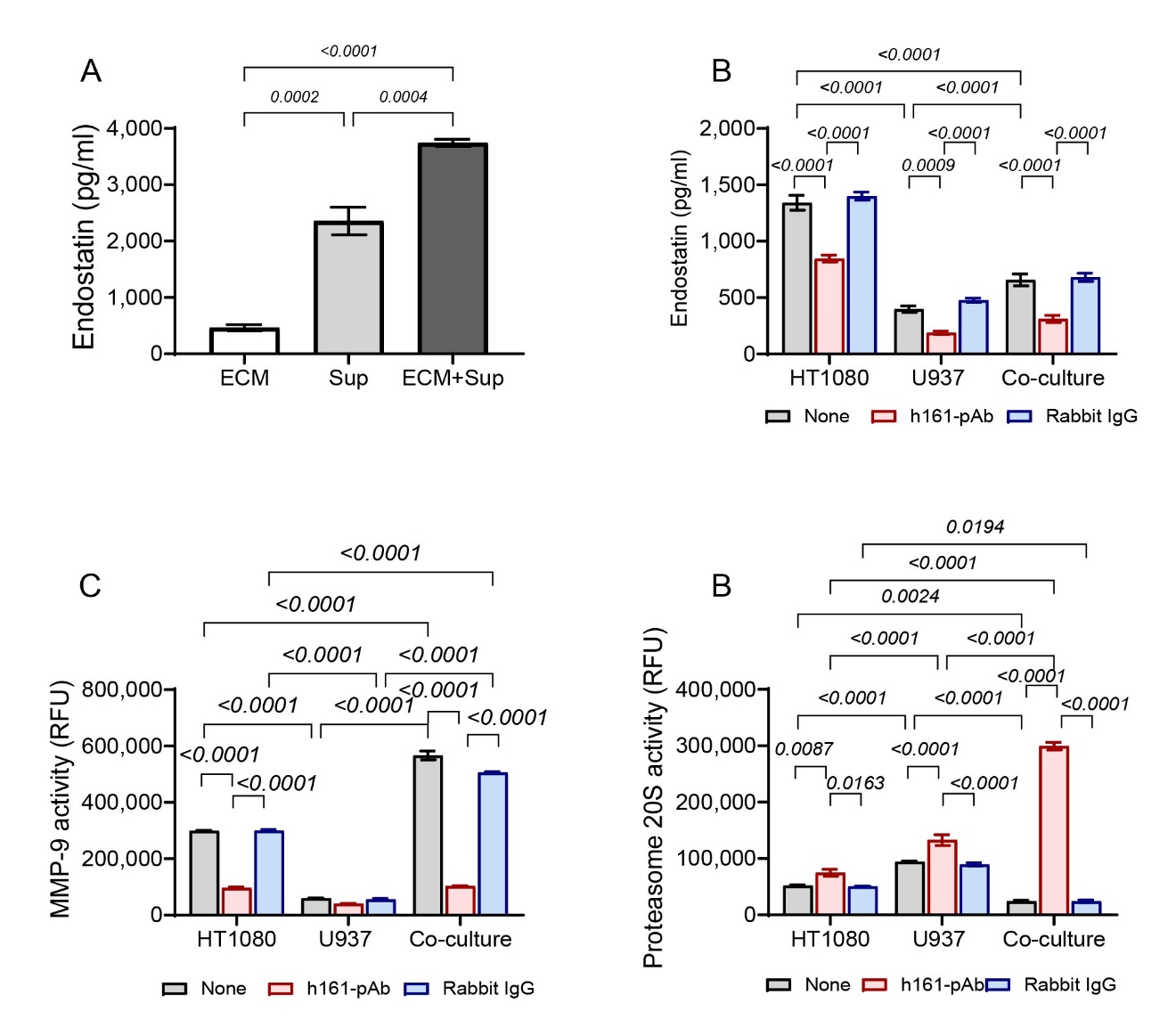
**

**Figure S3: Anti-CD147 antibody inhibits endostatin and MMP-9 activity, but enhances proteasome 20S activity outside the cells.** HT1080 (3x10^4^ cells) were incubated in serum starvation medium with TNFα (1ng/ml) for 72h to allow the cells to deposit their ECM proteins. Cells were then destroyed with double distilled water (DDW) for 20 min and cellular debris was washed away with PBS three times. Supernatants derived from the HT1080 and U937 single culture or their co-cultures were diluted 1:1 with fresh starvation medium, and allowed to digest the ECM for 48 h. At the end of the incubation, the supernatants were collected and **(A)** the concentrations of endostatin (n=6), **(B)** MMP-9 activity (n=6) and **(C)** the activity of proteasome 20S (n=6) were determined. Data are presented as means ± SE and analyzed using two-way ANOVA followed by Bonferroni's post hoc test.

**
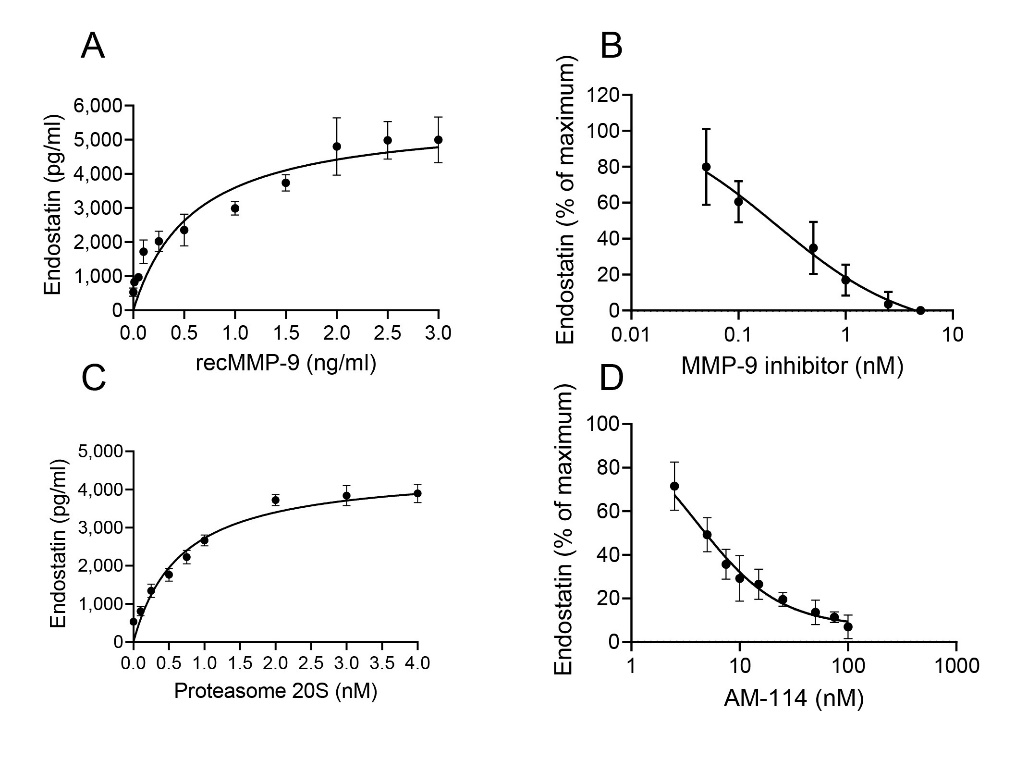
**

**Figure S4: Calibration of recombinant MMP-9, proteasome 20S and their respective inhibitors.** HT1080 (3x10^4^ cells) were incubated in serum starvation medium with TNFα (1 ng/ml) for 72h and allowed to deposit their ECM proteins. Cells were then destroyed with DDW for 20 min and cellular debris was washed away with PBS three times. The remaining ECM was incubated with increasing concentrations of **(A)** rec. MMP-9 that was activated by incubation with APMA (1 mM) or with **(B)** proteasome 20S that was activated with 0.05% SDS. Alternatively, the ECM was incubated with supernatants derived from the HT1080 cells, and increasing concentrations of **(C)** MMP-9 inhibitor I or **(D**) AM114 that were added for the 48h incubation. Endostatin levels were measured using ELISA. Data are presented as means ± SE (n=6).

**
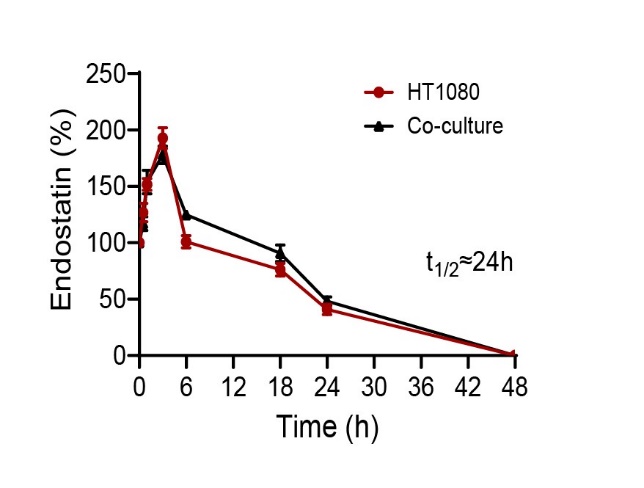
**

**Figure S5: Endostatin is degraded at the same rate in single and co-cultures.** To generate ECM, HT1080 (3x10^4^ cells) were incubated in serum starvation medium with TNFα (1 ng/ml) for 72h and allowed to deposit their ECM proteins. Then the cells were destroyed by incubating them for 20 min with double distilled water (DDW), and cellular debris was washed away with PBS three times. Supernatants derived from HT1080 cells, or their co-culture with U937 cells (3x10^4^ cells) were added to the ECM and allowed to generate endostatin for 24h. To stop new generation of endostatin, a mixture of MMP-9 inhibitor I (0.15 nM) and the proteasome 20S inhibitor AM114 (3 nM) was then added, and samples were obtained at different time points to measure endostatin concentrations (n=6). No difference was found in the rate of endostatin degradation between the single or co-cultures and the half-life of the protein was estimated at 24 h. Data are presented as means ± SE.

**
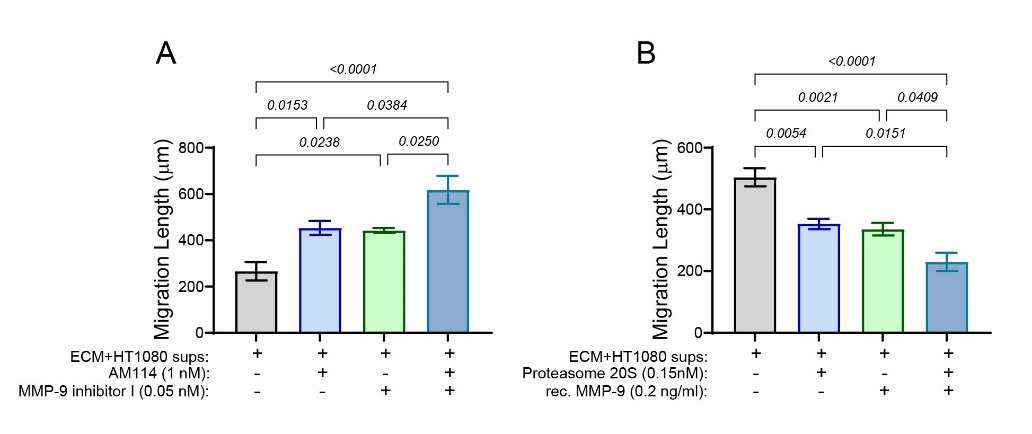
**

**Figure S6: MMP-9 and proteasome 20S activities and the resultant endostatin concentrations are manifested in the functional wound assay.** Supernatants were obtained from experiments where the ECM was incubated with the indicated concentrations of **(A)** AM114, MMP-9 inhibitor I or their combinations (n=6), or **(B),** the rec. MMP-9 or proteasome 20S (n=5) (as explained in the legend of Fig 7). These supernatants were applied unto a scratched monolayer of EaHy926 endothelial cells, as was described before. Data are presented as means ± SE and were analyzed using one-way ANOVA followed by the Bonferroni's post-hoc test**.**

**
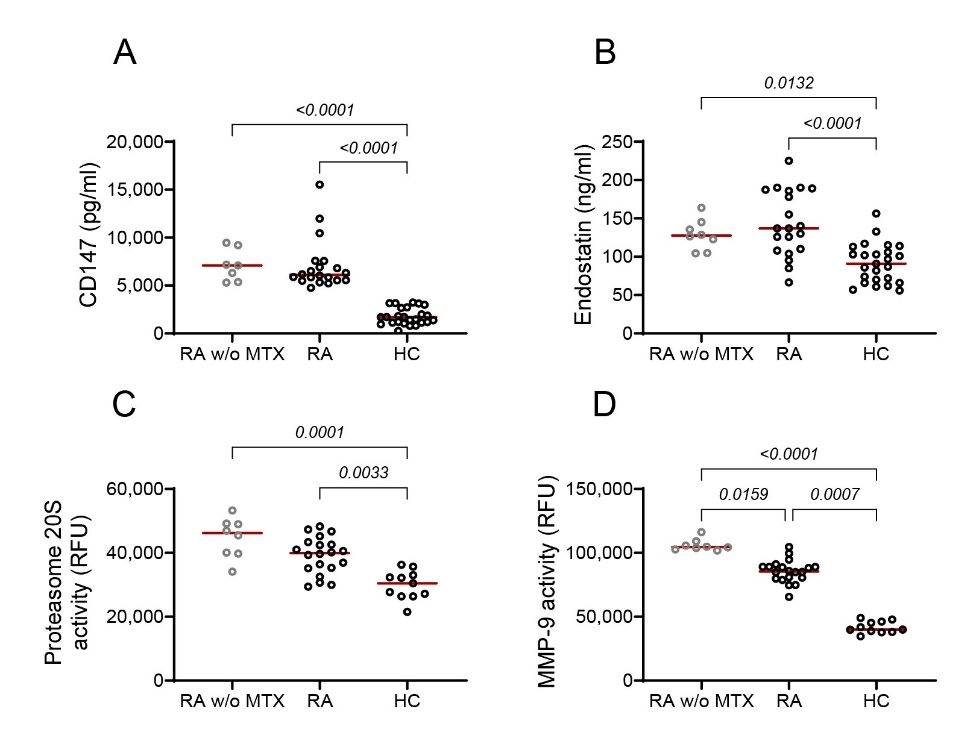
**

**Figure S7: The effect of methotrexate (MTX) on CD147, endostatin and the activities of MMP-9 and proteasome 20S.** Serum samples from patients with active RA that were treated with MTX (RA, n=23) or not treated with MTX or other cDMARDs (n=8), were examined for their **(A)** CD147 concentrations, **(B)** endostatin concentrations, **(C)** proteasome 20S activity and **(D)** MMP-9 activity. Data are presented as median values (red) and were analyzed using the non-parametric one-way ANOVA Kruskal-Wallis test followed by the Dunn's multiple comparisons test.
